# Self-Assembly and Cascade Catalysis by a Soft-Oxometalate (SOM) System

**DOI:** 10.3389/fchem.2020.601814

**Published:** 2020-11-27

**Authors:** Kousik Das, Tingting Yan, Shounik Paul, Shilun Qiu, Teng Ben, Soumyajit Roy

**Affiliations:** ^1^Eco-Friendly Applied Materials Laboratory, Department of Chemical Sciences, Indian Institute of Science Education and Research, Kolkata, India; ^2^Department of Chemistry, Jilin University, Changchun, China

**Keywords:** polyoxometalate, self-assembly, polymerization, cascade catalysis, oxidation

## Abstract

Cascade catalysis has gained importance due to its various applications. In this work, cascade catalysis was performed using a self-assembled soft-oxometalate (SOM) as a model system. At first, we synthesized an oxometalate (OM) hybrid with a polymerizable organic cation, namely tetrakis(4-aminophenyl)methane, and an OM, K_8_[SiW_11_O_39_]. The hybrid in turn was converted into SOM in water, DMSO mixture, and characterized by different techniques, ranging from electron microscopy to DLS. The SOM state is endowed with the ability to polymerize the aniline based counter ions associated with it in the presence of UV-light. This polymerization is possible due to the presence of photocatalytic OMs (oxometalates) in the SOMs. The polymer-SOM hybrid in cascade oxidizes selectively aniline to nitrobenzene and nitrite to nitrate owing to the residual oxidizing property of the OM constituents in it. This is the first example of cascade catalysis in SOM chemistry.

## Introduction

Assembly of multicomponent, multifunctional architecture is a challenge. For instance successful assembly of organic-inorganic hybrid material (Hagrman et al., 1999) leads to the emergence of non-linear unusual properties (Kagan et al., 1999; Yin et al., 2012, 2014; Dualeh et al., 2014). The achievement of a successful working design is thus an immediate challenge for chemists. As part of this process, questions emerge, as to whether it is possible to synthesize an inorganic-organic hybrid architecture based on oxometallate. Can such an assembled supramolecular architecture be functional? Here we address these questions, asking more specifically whether is it possible to design an organic moiety and oxometalate (OM) based self-assembly of a soft-oxometalate (SOM). Can such SOM be catalytically polymerized to form a SOM hybrid? Can the resulting SOM hybrid in turn show cascade catalysis? Before we answer these questions we explore the literature and explain our design.

Polyoxometalates (POMs), (Pope et al., 2004) crystalline state of transition metal oxo-clusters (oxometalates) have gained immense research interest due to their tuneable size and morphology, unique electronic properties, and wide range of applications like catalysis, electronics, nano materials, medicine etc. (Wang et al., 2003; Cronin and Müller, 2012; Lv et al., 2012; Wang and Weinstock, 2012). Due to the high oxidation states and photoexcitation properties of transition metals, polyoxometalates are very efficient and promising catalysts (Kikukawa et al., 2012; Suzuki et al., 2015; Wang and Yang, 2015). They have been used as catalysts for water splitting (Rausch et al., 2014), polymerization (Chen et al., 2013), and several other organic reactions over the past few decades. Our group has also published a series of catalytic reactions with oxometalates in recent years (Thomas et al., 2015; Das et al., 2016a,b,c). Oxometalates have a very unique solution behavior. Depending on their counter ions and volume fraction/concentration (Thomas et al., 2018), pH (Paul et al., 2018) oxometalates can show a heterogeneous/colloidal or soft-matter state, which has been called the soft-oxometalate (SOM) state (Roy, 2011, 2014). Owing to the intrinsic OM constituents in SOMs the latter holds promise to manifest the properties of OMs in higher length scales and bulk. SOMs thus provide a platform to manifest synergistic properties by the judicious assembly of large OMs (like PW_12_ Keggin, Mo_132_, (Verhoeff et al., 2007) Mo_154_ (Liu et al., 2003), and their counter-ions. To date, self-assembled SOMs have used simple counter-ions like protons, ammonium and alkaline earth metal ions. Such ions leave the chemistry of SOMs dormant. Hence the challenge in SOM-engineering necessitates the judicious choice of OM and counter-ions that can open up the possibilities of new SOM chemistry. If the counter-ions are reactive, redox polymerizable organic ligands like amino-phenyl methane with multiple sites, can open up a host of possibilities for self-assembly. This approach of combining molecular oxometalates with suitable organic moieties to make crystalline POM-organic hybrids has already been explored in the context of crystalline POM chemistry (Zhang et al., 2010; Chen et al., 2013). In this study we adapt this approach to SOM chemistry and demonstrate cascade catalysis for the first time.

As mentioned earlier, the choice of the OM and the organic counter ion in SOMs needs to be judicious. For instance, these units have to be mutually compatible to form SOMs and if the organic counter-ion is polymerizable then the resulting organic polymers can have direct applications (Heeger, 1993; Macdiarmid, 2001). In this regard, it is important to mention some synthetically important networks such as metal organic frameworks (MOF), (Long and Yaghi, 2009), covalent organic frameworks (COF) (Wan et al., 2009), and porous aromatic frameworks (PAF) (Ben et al., 2009; Ren et al., 2010; Xu et al., 2015), etc., which have a well-defined structure and high surface area. The high surface area of such frameworks leads to better catalyst loading and their rigid structure makes them excellent support for heterogeneous catalysis (Dong et al., 2016). Recently, conductive polymers attracted a great deal of interest because of their high electrical conductivity (Shumaila et al., 2011). Among the family of conducting polymers, polyaniline is one of the most exciting polymers, due to its unique electrical conductivity, high environmental stability, and easy synthesis.

For the past few decades, scientists have been synthetically modifying their structure to use them in more commercial applications. There are several techniques of polymerization of such aniline based monomers of which redox polymerization (Kohut-Svelko et al., 2009) is worth mentioning in the present context, as it can utilize a redox-active OM center that can be built in the SOM. This redox polymerization is facile, attractive, and has a short induction period attracting mild conditions that are conducive to SOM chemistry (Zengin et al., 2002; Fehse et al., 2007).

**Figure 1:**
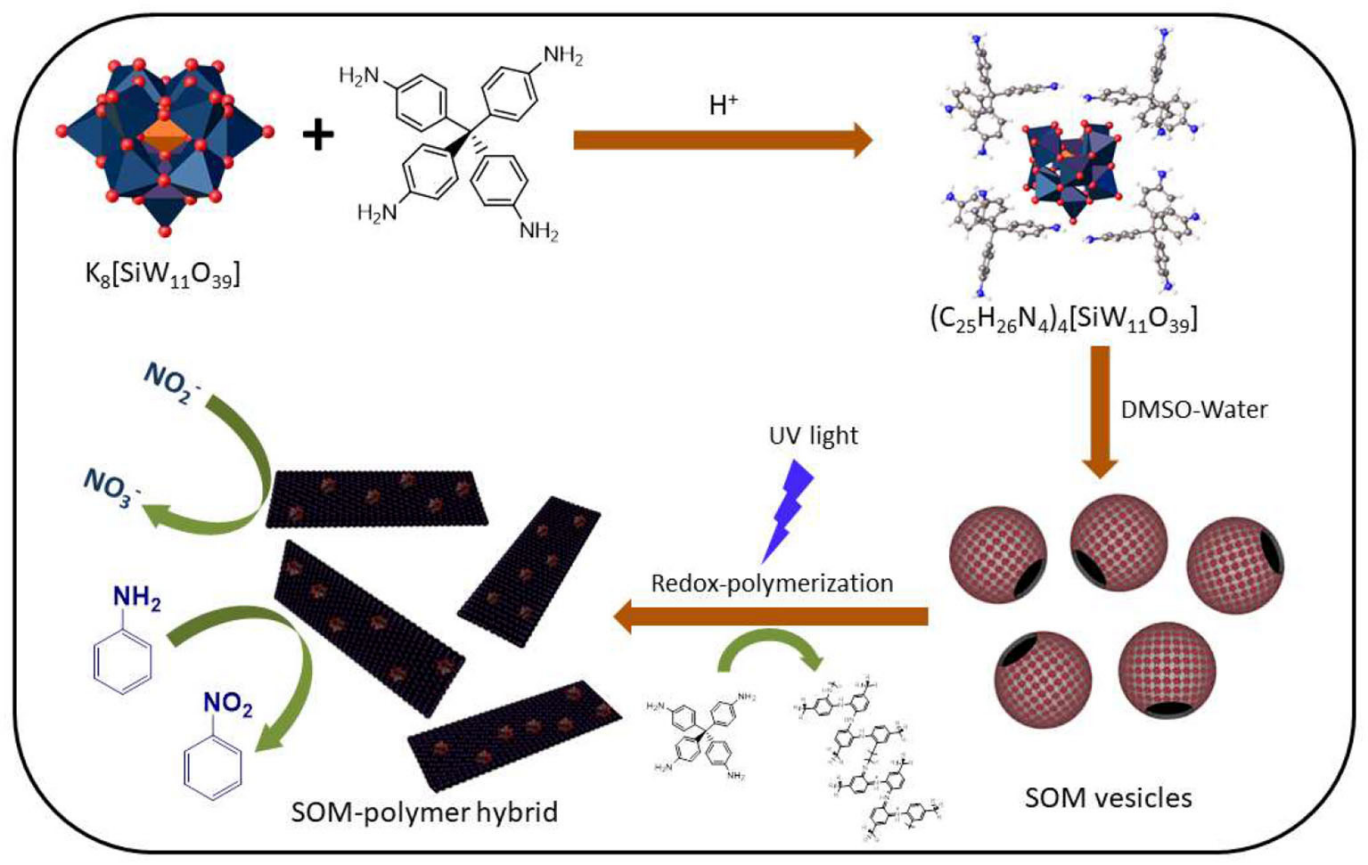
Schematic image of the preparation of (C_25_H_26_N_4_)_4_[SiW_11_O_39_] and the polymerization of the organic counter-ion thereafter by silicotungstate.

As mentioned earlier, various methods for the syntheses of oxometalate-organic hybrids have been reported in the literature (Bar-Nahum et al., 2003; Dolbecq et al., 2010). However simple organic ligands have been used in most of these methods and there are very few reports on the reactivity of their side chains (Rieger et al., 2012; Lachkar et al., 2016). There are still questions as to whether it is possible to synthesize an OM-organic hybrid that can catalyze a reaction on its side chain *in-situ*. After assembling the molecular hybrid, is it possible to superstructure the architecture as a SOM? Can the resulting SOM state have catalytic properties? This study addressed these questions by synthesizing a crystalline polyoxometalate-organic hybrid from a photo-redox active lacunary Keggin K_8_[SiW_11_O_39_] and tetrakis(4-aminophenyl)methane (Uribe-Romo et al., 2009; Liu et al., 2014) with a redox polymerizable aniline side chain which can grow to form a three dimensional polymeric network, and stabilize itself as a SOM and SOM-polymer hybrid. The SOM-polymer hybrid in turn, in the fashion of cascade catalysis, electrochemically oxidizes nitrite to nitrate and aniline to nitrobenzene. Thus, the assembly and cascade catalysis reported in this work has the following steps. First, the assembly of OMs with aniline based counter ions to SOMs; second, the catalytic formation of SOM-polymer hybrid; and third, a cascade catalytic conversion of aniline to nitrobenzene and nitrite to nitrate (Figure 1).

## Results and Discussion

This study initially focused on the synthetic aspects of the SOM, using the following steps to investigate the reaction. First, the crystalline molecular POM-organic hybrid is synthesized and self-assembled to SOMs. Then the hybrid is polymerized. Using this polymerized hybrid, cascade catalysis was then performed.

### Synthesis and Characterization of OM-Organic Hybrid

**Figure 2:**
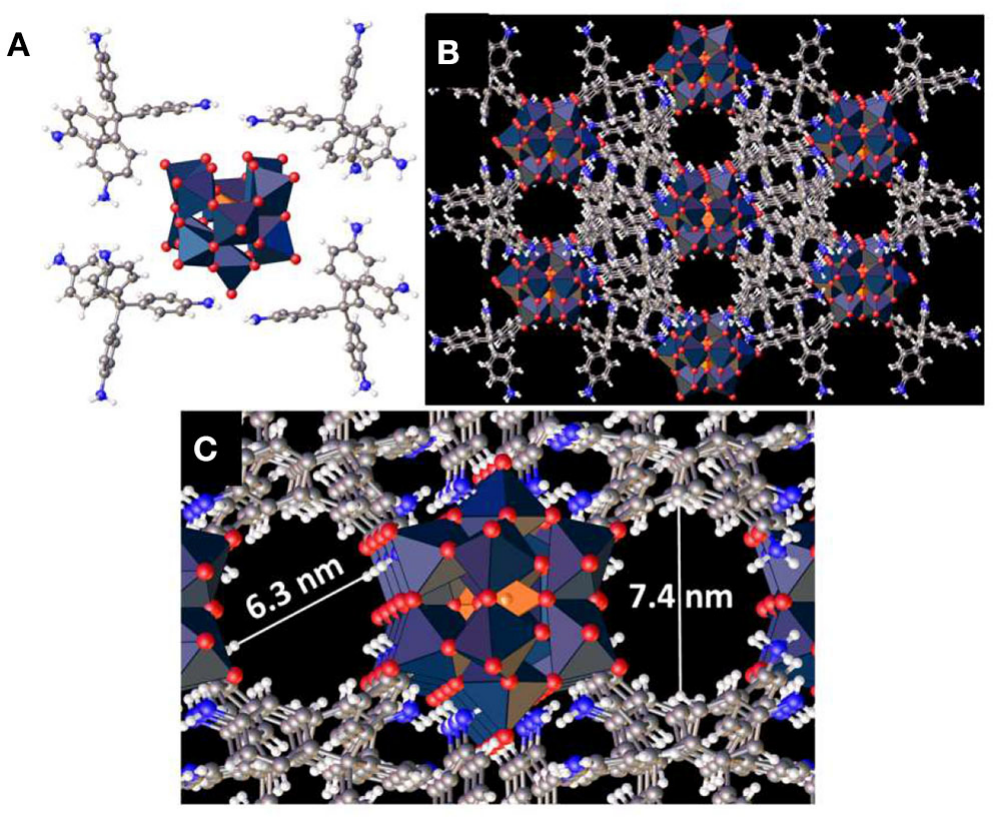
**(A)** Crystal structure of (C_25_H_26_N_4_)_4_[SiW_11_O_39_]. **(B,C)** 3D structure of the hybrid showing pores inside the framework.

The synthesized crystalline polyoxometalate hybrid has two components: tetrakis(4-aminophenyl)methane and [SiW_11_O_39_]^8−^. To gain molecular structural insights on this hybrid we crystallized the hybrid (Figure 2A). The structure of the model compound shows that each of the oxometalate units is connected to 8 ammonium ions. It is also evident from the structure that in each organic moiety only two of the four –NH_2_ groups are protonated. The crystal structure also shows two different cavities with a diameter of 6.3 and 7.4 nm (Figures 2B,C). This is due to the presence of asymmetric oxometalate units in [SiW_11_O_39_]^8−^. These cavities are very important for the stabilization of organic oligomers and the oxometalates present around these cavities facilitate the polymerization reaction that leads to the formation of these oligomers.

**Figure 3:**
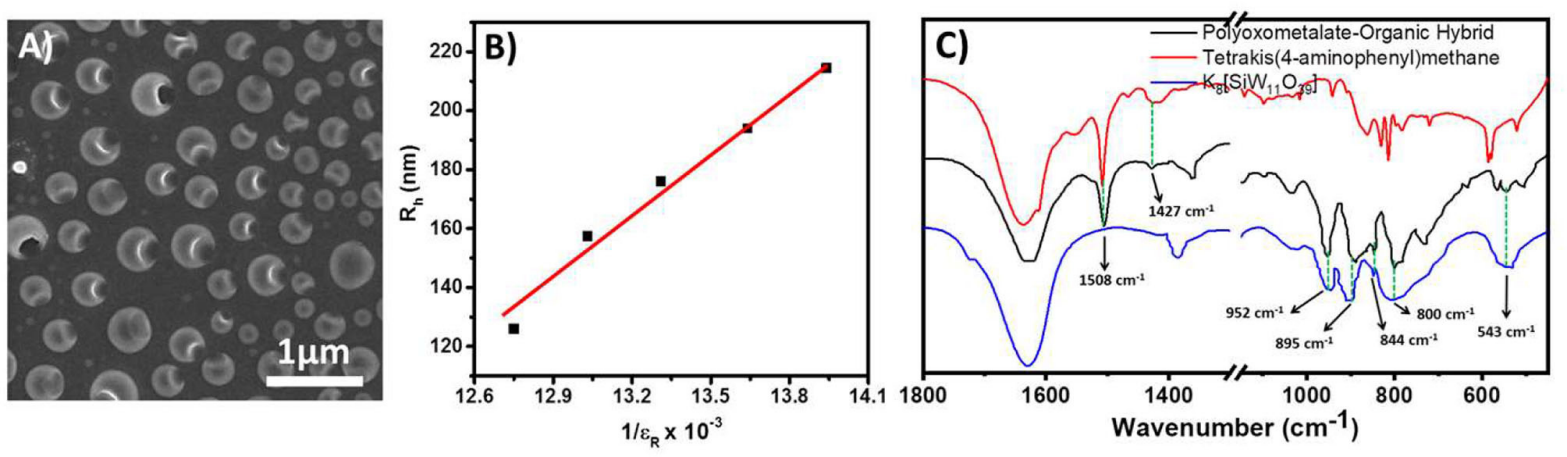
**(A)** SEM image of the hollow vesicles of the hybrid prepared in water, DMSO mixture. **(B)** Plot of soft-oxometalate vesicle radius with respect to the inverse dielectric constant of the solvent mixture. **(C)** Comparison of IR spectra of oxometalate hybrid, tetrakis(4-aminophenyl)methane, and [SiW_11_O_39_]^8−^.

This molecular oxometalate-organic hybrid further manifests in a dispersion soft-oxometalate structural state as vesicles. We assume that the hybrid retains its solid-state molecular architecture in the dispersion (soft-matter state) that facilitates the polymerization reaction in the cavity (Chen et al., 2013). To further analyze the structural integrity of oxometalate as well as the organic ion, we studied the FTIR spectrum of the hybrid. The following characteristic peaks of [SiW_11_O_39_]^8−^, 952, 895, 844, and 800 cm^−1^ were found in hybrid oxometalate whereas the characteristic peaks of tetrakis(4-aminophenyl)methane were found at 1,508, 1,427 cm^−1^ (Figure 3C).

### Preparation of Soft-Oxometalate From the Hybrid

We observed that the molecular hybrid shows an amphiphilic character. It can be easily dissolved in DMSO to make a stable dispersion. A relatively low scattering intensity in the dispersion confirms the absence of any large assemblies and the hybrid remains as a discrete molecular species in solution. Upon the addition of water into the DMSO solution, the solution becomes turbid, which suggests the formation of vesicle like SOM structures in the dispersion. We have characterized the vesicles by DLS and SEM analysis. The hydrodynamic radius (R_h_) was measured by DLS (Supplementary Figure 3). At 1:9 DMSO water ratio, the vesicle size appears to be ca. 160 nm. In the hybrid structure, the [SiW_11_O_39_]^8−^ unit acts as the hydrophilic part and the organic counter ion tetrakis(4-aminophenyl)methane acts as the hydrophobic part. Since the water content is more in dispersion, we believe that in the structure the oxometalate remains on the outer surface whereas the organic part remains on the inner surface.

The size of SOM vesicles was then varied by changing the solvent polarity (Verhoeff et al., 2007). The polarity of the solvent was varied by mixing different amounts of water and DMSO. We found that the vesicle size displayed a linear relationship with the inverse of the dielectric constant of the solvent (Figure 3B). This implies that the vesicle size can be varied by controlling the dielectric constant of the medium, implying counter ion condensation stabilizing the dispersion.

**Figure 4:**
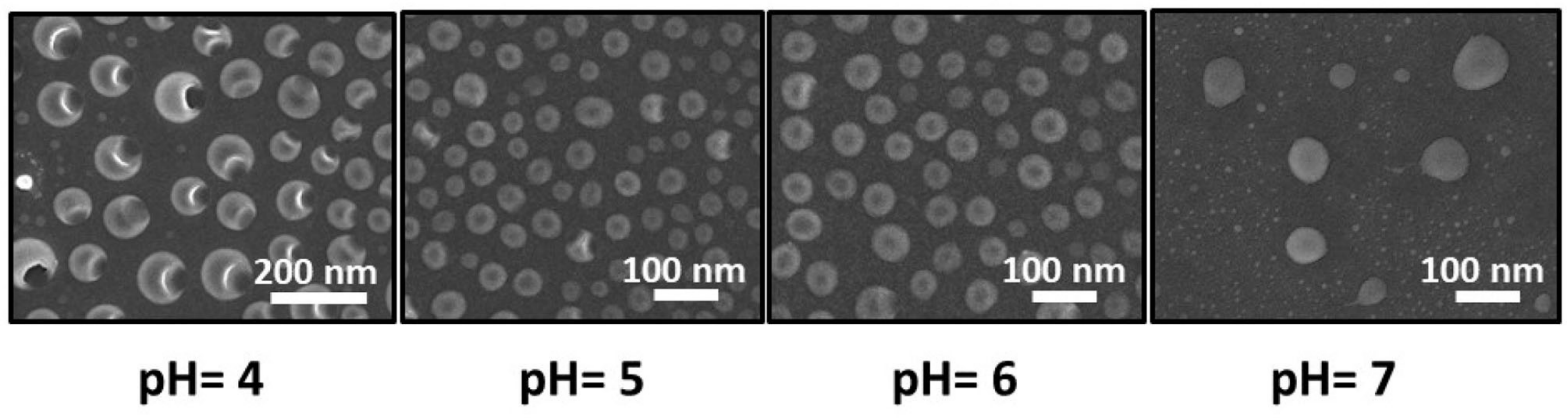
Shape change of the vesicles in different pH. Porous vesicles were observed at ~pH = 4.

The spherical nature of the vesicle was characterized by SEM analysis. The sizes obtained from SEM images are also consistent with the LS analysis (Du and Chen, 2004; Verma et al., 2005; Leng et al., 2010). Each of the vesicles shows large pores on the surface (Figure 3A) at pH 4. We believe that this is due to disruption of the surface during the drying process. However, upon adding a base, the pore(s) on the vesicle surface did not appear (Figure 4) (Sandre et al., 1999). The pK_b_ of aniline moiety was 9.4. At low pH (4.6) the amine group remained at an equilibrium between –NH_2_ and –${\text{NH}}_{3}^{+}$. Hence the surface of the vesicle was much more labile. With the addition of a base, the pH of the solution increases, and thus the amine groups mainly remained in –NH_2_ form and it was less prone to disrupt the hard skin of the surface during the drying process (Liu et al., 2005; Jeong et al., 2007).

**Figure 5:**
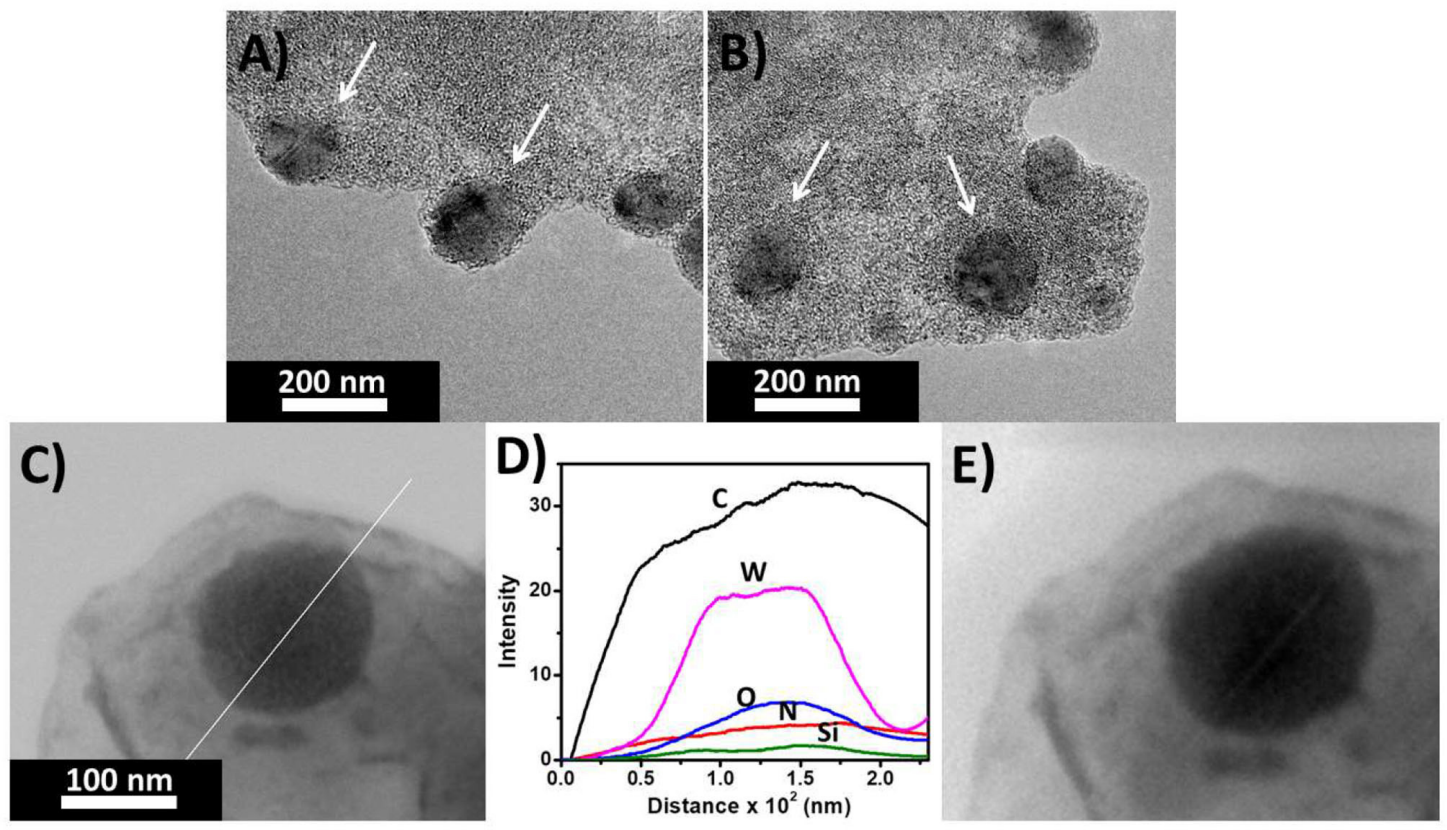
**(A,B)** TEM image of the SOM vesicles of (C_25_H_26_N_4_)_4_[SiW_11_O_39_] **(C)** Line scan image and **(D)** plot of intensity of different elements along the line. **(E)** Beam-damaged image of the hybrid after line scan.

**Figure 6:**
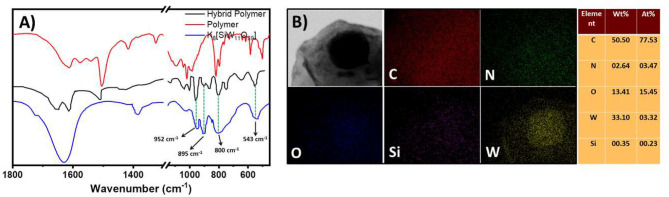
**(A)** Comparison of IR spectra of the polymer, OM-polymer hybrid, and K_8_[SiW_11_O_39_]. **(B)** EDAX mapping and elemental percentage of the (C_25_H_26_N_4_)_4_[SiW_11_O_39_] hybrid. C, N, O, Si, and W represent carbon, nitrogen, oxygen, silicon, and tungsten, respectively.

From the TEM we observed that a SOM hybrid was formed with tetrakis(4-aminophenyl)methane and [SiW_11_O_39_]^8−^ (Figure 5). The topology is spherical with a uniform molecular level distribution of elemental Si, W, O, N in the mesoscopic SOM structure (Figure 6B). We believe that the positively charged ammonium ions behave as an anchor for attaching the negatively charged oxometalates via electrostatic interaction.

### *In-situ* Polymerization of the Hybrid

We then explored whether the hybrid possessed any catalytic properties. Oxometalates are well-known for their photocatalytic properties. Recently, we photo-polymerized monomers like styrene, and acrylic acid, etc., with PW_12_ based polyoxometalate in the presence of UV radiation. This approach polymerizes the monomers via cation-radical polymerization. In the present case, the [SiW_11_O_39_]^8−^ also catalyzes the polymerization reaction of the tetrakis(4-aminophenyl)methane monomer in presence of UV-light (Supplementary Figure 4). The structure of the hybrid also plays an important role in polymerization. The solid state structure of the hybrid has cavities in it. We believe the hybrid retains its solid state structure in the dispersion (soft-matter state) through the formation of soft-oxometalate. We have previously shown that these cavities stabilize the monomer and facilitate the formation of the polymer (Chen et al., 2013). In this case, also, we believe that the monomers are stabilized in the structural cavity and the oxometalates present in the cavities polymerize the monomers via photoredox polymerization. Tetrakis(4-aminophenyl)methane has four aniline units in its structure and can be polymerized by an oxidation reaction. The polymerization of the monomer happens in 2 weeks without any catalyst but in presence of oxometalate, the polymerization reaction happens in just 8 h (Supplementary Figure 8).

### Characterization of the Polymer

The FTIR study showed several new peaks (985, 1,215, 1,322, and 2,576 cm^−1^) after polymerization. The peaks ranging from 1,570 to 1,600 cm^−1^ occur because of the C=N stretching of the quinoid structure (Figure 6A) (Mostafaei and Zolriasatein, 2012).

^1^H and ^13^C NMR analysis of the polymer also showed several changes in the spectra, which confirms the polymerization of the monomer (Supplementary Figures 10–16). The peaks ranging from 6 to 8 ppm in the ^1^H spectrum are due to the aromatic protons present in the polymeric structure (Supplementary Figure 11) (Yasuda and Shimidzu, 1993). The peaks at 148 and 150 ppm in ^13^C NMR also confirm the presence of the quinoid structure of the polymer (Supplementary Figure 16). The H^+^ ions present in the solution attach with the nitrogen atoms of the polymer to form ammonium or iminium ions. We believe that this is because the polymer is still charged (ammonium or imminium ion) and the oxometalate remains electrostatically attached to the polymer. This is also evident from the FTIR spectrum of the polymer, which shows both the peaks of the polymer as well as the silicotungstate ion in the resultant polymer hybrid (Figure 6A).

**Figure 7:**
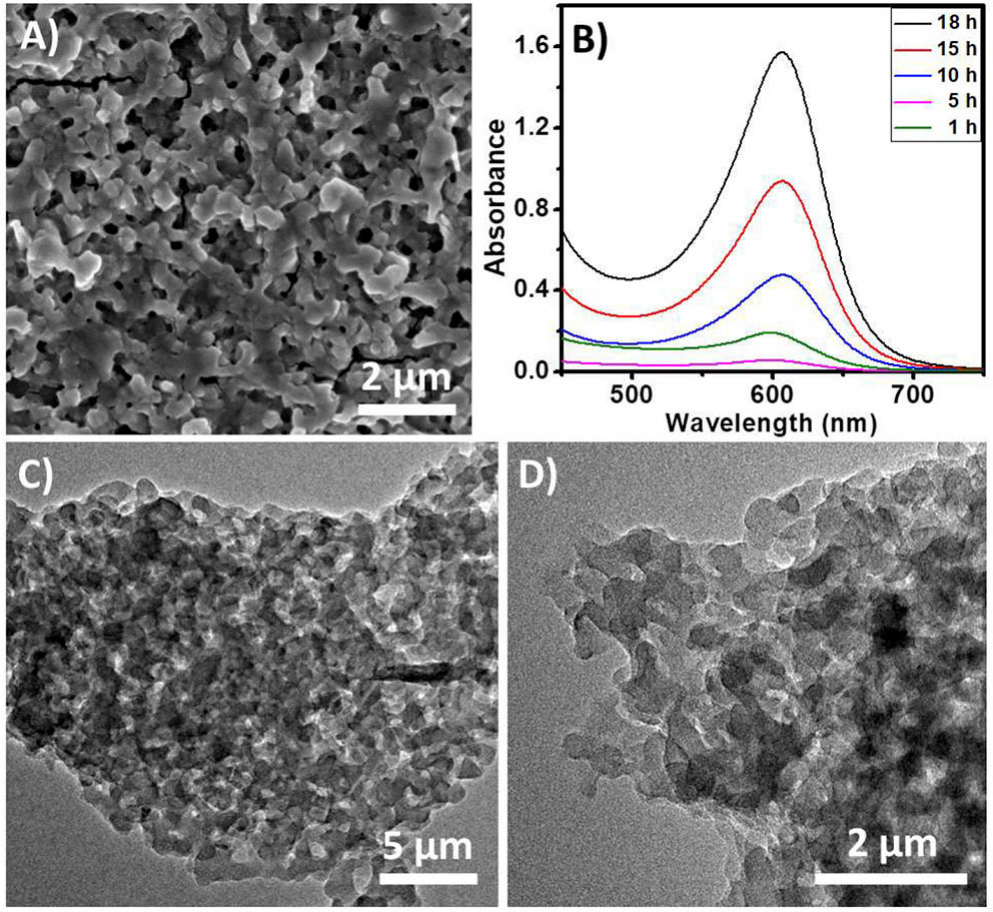
**(A)** SEM image of the polymer prepared from the hybrid. **(B)** Time dependent EAS of the polymer in DMSO. **(C,D)** TEM images of the SOM-polymer hybrid.

**Figure 8:**
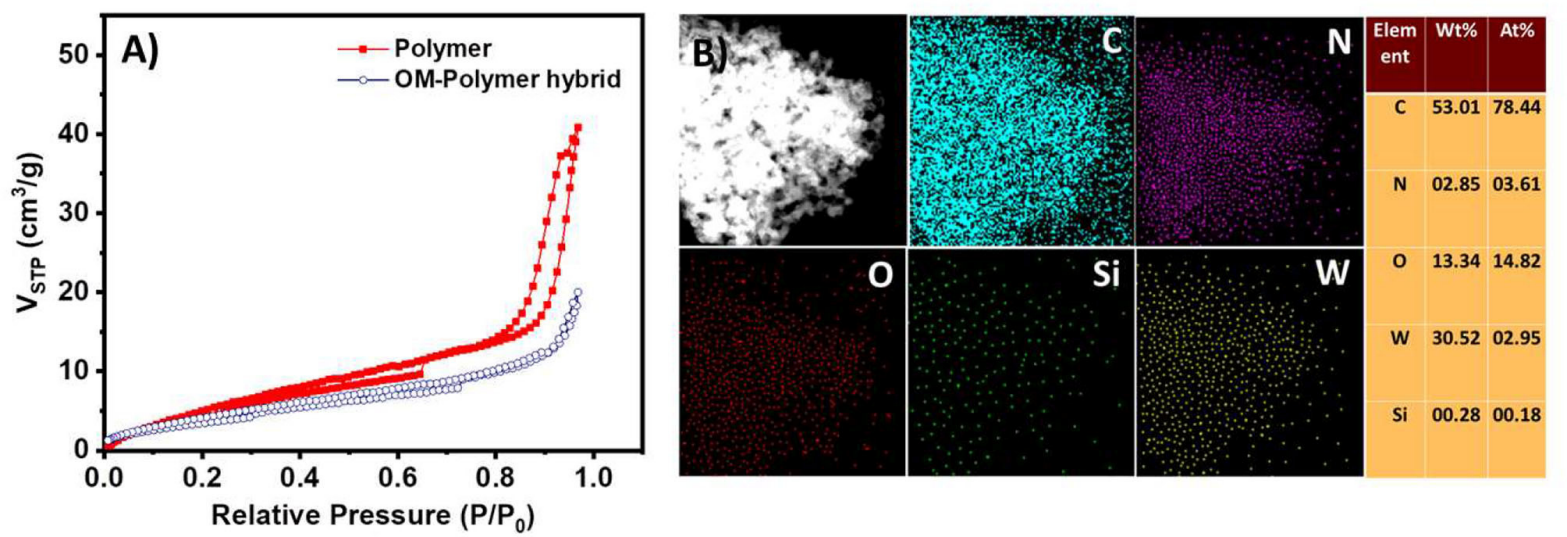
**(A)** N_2_ adsorption–desorption isotherms of polymer and SOM-polymer hybrid. **(B)** EDAX mapping and elemental percentage of the SOM-polymer hybrid. C, N, O, Si, and W represent carbon, nitrogen, oxygen, silicon, and tungsten, respectively.

The EAS of the polymer shows a peak at 600 nm (Figure 7B). This peak corresponds to the blue color of the solution. These observations also suggest that the polymer has an emeraldine structure. The absorption band at 600 nm corresponds to the n-π^*^ transitions of the quinine-imine groups (Yasuda and Shimidzu, 1993; Sapurina and Stejskal, 2008; Liu et al., 2010; Sapurina and Shishov, 2012). The band intensity increases with time, which suggests the formation of more polymeric structures. SOM-polymer hybrid has also been characterized by SEM and TEM (Figure 7). From the EDAX mapping of the hybrid (Figure 8B), we observed that the OMs are homogeneously distributed on the polymer surface. To locate the oxometalates in this hybrid polymer, we used porosity measurements. The BET surface areas of the chemically synthesized polymer and that of the SOM-Polymer hybrid are 19 and 14 m^2^/g, respectively (Figure 8A). We believe that some of the pores present in the polymer are filled by the oxometalates and hence the measured surface area is less in the polymer-oxometalate hybrid.

This study then addressed whether it is possible to change the chain length of the polymer. The chain length variation was achieved by changing the loading of oxometalate in the dispersion. The same amount of monomer was dissolved in five reaction vessels and then a different amount of OM was added to it. The reaction vessels were kept for 1 day to polymerize. The polymers were then separated from the solution and their chain length and molecular weight were measured by viscometry (Supplementary Table 2). The empirical rule of Mark-Houwink relation was used to measure the molecular weight of the polymers (Rana et al., 2011). We observed that the molecular weight, as well as the chain length of the polymer, decreases with the increased loading of oxometalate in the starting SOM (Table 1). The plot of the molecular weight of the polymer and loading of oxometalates shows a linear decrease (Supplementary Figure 9). This indicates that the polymerization happens via radical pathway. To ensure the operation of the radical pathway in the reaction, we added catechol to the reaction mixture as a radical quencher and the polymerization reaction did not take place in the presence of catechol.

**Table 1:** Chain length variation of the polymer by changing the amount of catalyst.

**Sample no**.	**Amount of OM (mmol)**	**Amount of monomer (mg)**	**Molecular weight (kDa)**
1	0.4	1	14
2	0.6	1	9.5
3	0.7	1	8.2
4	0.9	1	6.9
5	1.0	1	1.5

The chain length variation of the polymer is also evident from the different colors of the polymers dissolved in DMSO (Supplementary Figure 7B). This was because, as the chain length varies, the number of monomer units vary, and hence the degree of conjugation also varies. The different extent of conjugation in the different polymers leads to different colors of the solution. In electronic absorption spectra (EAS), the intensity of the 578 nm peak also decreases with the increase in polymer chain length (Supplementary Figure 7A). This is due to the presence of fewer polymer units in higher chain length polymers. The peak at 460 nm in EAS suggests the formation of the emeraldine of the polymer. We think that with the increase in OM concentration, the acidity of the solution increases, and hence more imine nitrogen gets protonated to give emeraldine salt of the polymer.

### Oxidation of Aniline to Nitrobenzene

The selective oxidation of aniline to nitrobenzene is very important in industrial synthesis. The oxidation of aniline leads to several products namely nitrosobenzene, nitrobenzene, azoxybenzene, azobenzene, etc. (Zhu and Espenson, 1995; Priewisch and Rück-Braun, 2005; Tundo et al., 2008; Shiraishi et al., 2014). Although there are various reports in the literature involving aniline oxidation, very few have reported selective oxidation toward nitrobenzene (Meenakshi et al., 2018). Even some polyoxometalates as well as polyoxometalate-organic hybrids have been employed for the oxidation of aniline, although they require harsh reaction conditions and the catalyst has low selectivity and low recovery.

**Figure 9:**
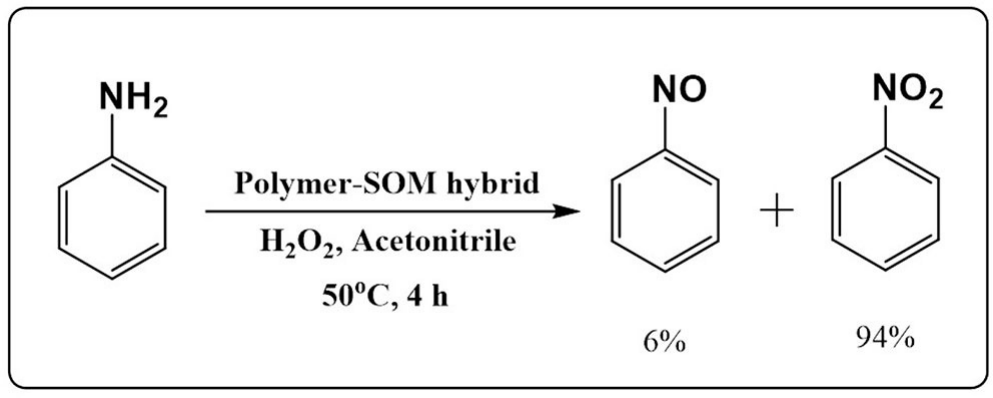
Schematic of aniline oxidation by SOM-polymer hybrid in acetonitrile.

In this article, we have utilized the polymer-SOM hybrid as a catalyst for the oxidation of aniline (Figure 9). Due to the presence of redox active W centers and a robust support structure of the polymer network, the polymer-SOM hybrid acts as an excellent catalyst. The reaction was performed in the presence of H_2_O_2_ at 50°C. In this reaction condition, the polymer-SOM hybrid oxidizes aniline selectively to nitrobenzene. A negligible amount of nitrosobenzene was also detected. All the products were detected in gas chromatography (Supplementary Figure 5). To check the role of solvent, the reaction was carried out in different solvents (Supplementary Table 1). The catalyst was recovered after each catalytic cycle (Supplementary Figure 6). The highest yield was detected in acetonitrile and lowest in H_2_O. It should also be noted that a very good yield was also detected in DMSO. However the recovery of the catalyst is low compared to other solvents. An increase in reaction temperature, increases the reaction rate, although a small amount of azoxybenzene was also detected at a higher temperature. At lower temperatures, the reaction rate was comparatively slow. Hence the optimum temperature was set to 50°C. In contrast, the polyoxometalate (K_8_[SiW_11_O_39_]) has less conversion efficiency, and selectivity is also very low in the same reaction condition. It is also worth mentioning that the (K_8_[SiW_11_O_39_]) is very unstable and thus has low recovery (Table 2).

**Table 2:** Comparison of aniline oxidation^a^ to nitrobenzene by different catalysts.

**Catalyst**	**Total conversion (%)**	**Products (%)**		**Recovery**
		**Nitrosobenzene**	**Nitrobenzene**	
SOM-polymer	96	6	94	91
K_8_[SiW_11_O_39_]	71	78	22	48

a *Aniline (1 mmol), 30% H_2_O_2_ (3 mmol), acetonitrile (5 mL), catalyst (5 mg), 50°C, stirred for 4 h*.

By definition, a cascade reaction comprises two steps where the second reaction happens because the chemical change occurs in the first step. In our case, the first step is the polymerization of tetrakis(4-aminophenyl)methane by [SiW_11_O_39_]^8−^ in the presence of UV light. The SOM in-turn transforms into an SOM-polymer hybrid. The second step is the catalysis reaction performed by the SOM-polymer hybrid. The SOM-polymer can oxidize nitrite to nitrate electrochemically or selectively oxidize aniline to nitrobenzene thermally. In both cases, the catalysis does not occur with the SOM or the monomer and oxometalate alone. This is where cascade catalytic property emerges due to the presence of various interactions in the SOM-polymer hybrid. The polymeric network forms during the first photocatalytic experiment and endows the SOM-polymer with an emergent catalytic activity that manifests during the aniline oxidation and carries out the concluding steps of cascade catalysis during nitrite oxidation. Without the polymeric network, the OM ([SiW_11_O_39_]^8−^) has less stability, low conversion, and poor selectivity toward aniline oxidation.

To check the stability of the catalyst we have performed IR and EAS spectroscopy. After the reaction of aniline oxidation, the recovered catalyst was thoroughly washed with ethanol and water and dried over vacuuo. For IR spectroscopy the powdered catalyst was used, whereas the catalyst was dissolved in DMSO for EAS analysis. Both IR and EAS the spectra of the recovered catalyst did not show any significant changes (Supplementary Figures 17, 18). This confirms that there was no leaching of OMs during the catalysis. The polymer network that is present in the hybrid prevents deterioration of OMs and provides the stability during oxidation of aniline.

### Electrochemical Measurements

**Figure 10:**
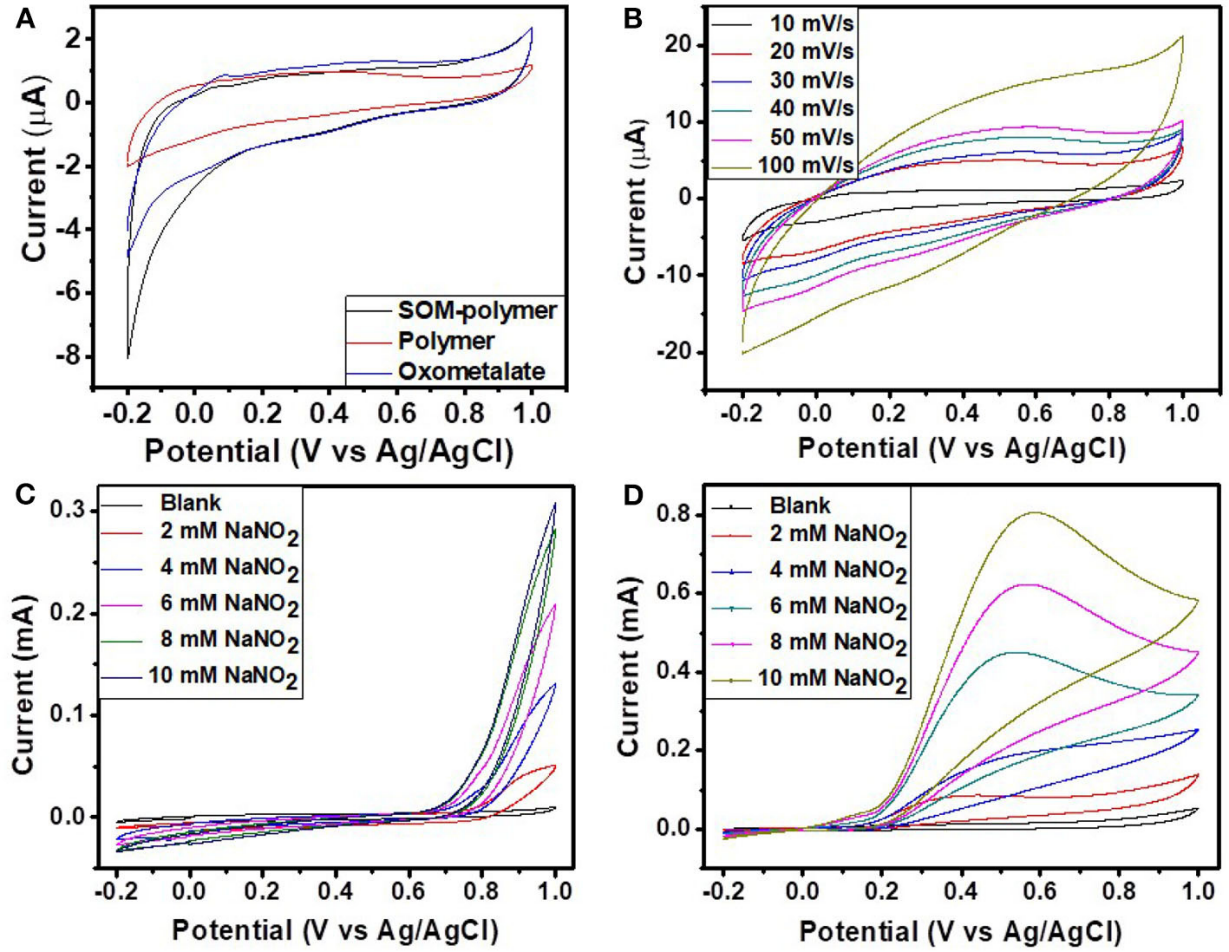
**(A)** The comparison CVs of SOM-polymer, pure polymer, and pure oxometalate at a scan rate of 10 mV/s. **(B)** The CVs of the SOM-polymer hybrid at different scan rates ranging from 10 to 100 mV/s. CVs of **(C)** pure polymer and **(D)** SOM-polymer hybrid with different amounts of NaNO_2_. All of the electrochemical measurements were done in 0.5 M H_2_SO_4_ using Ag/AgCl as a reference electrode.

The electrochemical properties of the hybrid materials were measured in 0.5 M H_2_SO_4_ by cyclic voltammetry using the three electrode system. Cyclic voltammetry was carried out in a potential range between −0.2 and 1 V at different scan rates ranging from 10 to 100 mV. Figure 10A represents the comparative CVs of different hybrid materials. The CV of the SOM-polymer hybrid shows the characteristic peaks of pure polyoxometalate. The cyclic voltammogram of the pure polymer does not show any noticeable activity. Figure 10B represents the CVs of SOM-polymer hybrid at different scan rates (from 10 to 100 mVs^−1^). The cathodic and anodic peak current as well as peak separation potential gradually increase with increasing scan rate. The electrochemical stability of the OM-polymer hybrid was also measured. The OM-polymer hybrid electrode was studied under a continuous cycle with a scan rate of 50 mVs^−1^ for 100 cycles. The cathodic and anodic peaks show very little change after the 100th cycle, which indicates very good stability of the hybrid. Compared to the hybrid, pure oxometalate is unstable and easily washed out during the electrochemical measurements.

We then explored whether it is possible to use this hybrid as a catalyst for an important oxidation reaction, the nitrite oxidation reaction. Nitrite is toxic to the human body. It forms nitrosamines and subsequently diazonium ions. The reactive diazonium ions disrupt normal cell function which leads to cell death. Nitrite can be detected by oxidation as well as reduction reaction. Though the reduction of nitrite is a little complicated, the oxidation reaction is pretty straight forward, which produces the only nitrate. Polyoxometalates are well-known for their catalytic activity. There are numerous reports of nitrite oxidation being performed by different catalytic materials but the question remains as to whether the SOM-polymer hybrid electrochemically catalyzes this reaction. In this article, we investigated nitrite oxidation using the SOM-polymer hybrid as a catalyst.

Nitrite oxidation of the SOM-polymer hybrid was performed in 0.5 M H_2_SO_4_, applying the potential range of −0.2 V to 1 V at a scan rate of 50 mVs^−1^. Figure 10D is a cyclic voltammogram of nitrite oxidation reaction with SOM-polymer hybrid at different concentrations of nitrite ranging from 2 to 10 mM. The oxidation peak for the SOM-polymer hybrid was found at around 0.57 V with respect to the Ag/AgCl reference electrode, though peak shift was observed for different concentrations of NaNO_2_. The SOM-polymer hybrid showed an increase of 0.65 mA peak current for 10 mM NaNO_2_ (Figure 10D). For a comparative study, we also performed the oxidation reaction with the pure polymer and pure polyoxometalate. There was no oxidation peak found in the same region for the polymer (Figure 10C) and negligible nitrite oxidation was observed for the pure polyoxometalate. The (C_25_H_26_N_4_)_4_[SiW_11_O_39_] also shows activity toward nitrite oxidation, though the current is not as high as in a polymer-SOM hybrid (Supplementary Figures 1, 2). This observation implies the emergence of catalytic activity of the SOM-Polymer hybrid that in a cascade catalyzes nitrite oxidation.

## Conclusions

To conclude, this study successfully grafted a polymerizable organic ion onto an oxometalate. The structure of the molecular hybrid was confirmed by the SCXRD pattern and the hybrid forms SOM in water DMSO mixture. The composite SOM, by the virtue of having a redox active oxometalate, polymerizes the side chain of tetrakis(4-aminophenyl)methane into a polymer. We characterized the polymeric structure by IR as well as NMR spectra, thus creating a system for a polymer-colloid (SOM) mixture. This can be used for nitrite oxidation and the selective oxidation of aniline in a cascade catalytic mode. Thus in short we show first a self-assembly of an organic-inorganic hybrid SOM. By then exploiting the intrinsic redox property and acidity of the contained oxometalate in SOM, we oxidized the aniline based side chain to polyaniline. This polymer-SOM composite, in turn, owing to its intrinsic residual redox property, oxidizes aniline to nitrobenzene selectively, and electrochemically oxidizes nitrite to nitrate. We have thus shown, for the first time, cascade catalysis in the context of SOM chemistry, which could further explorations in the context of sustainable chemistry.

## Data Availability Statement

The datasets generated for this study can be found in online repositories. The names of the repository/repositories and accession number(s) can be found at: CCDC, 1580136.

## Author Contributions

KD, TY, and SP performed all the experiments and collected the data. SR conceived and designed the experiments and wrote the paper with input from all authors (particularly KD). Part of the experiments was performed in SR's laboratory and part in the facilities of TB and SQ. All authors read and approved the paper.

## Conflict of Interest

The authors declare that the research was conducted in the absence of any commercial or financial relationships that could be construed as a potential conflict of interest.
