# Supplementary material for: Self-Assembly and Cascade Catalysis by a Soft-Oxometalate (SOM) System
Source: Front Chem. 2020 Nov 27;8:601814. doi: 10.3389/fchem.2020.601814 (PMC7729020; doi:10.3389/fchem.2020.601814)

Supplementary Material

Experimental Section

Materials

All chemicals were purchased from commercially available sources and used without purification unless otherwise stated. Double distilled water was used for all the experiments. All the glassware were washed properly with water and acetone and dried in oven before use.

Preparation of oxometalate-organic hybrid

In a 100 mL beaker 400 mg (1.05 mmol) of tetrakis(4-aminophenyl)methane was dissolved in 20 mL of 1 M HCl solution. 1 g (0.35 mmol) of $K_8[SiW_{11}O_{39}]$ was slowly added to the solution with vigorous stirring. After the addition of $K_8[SiW_{11}O_{39}]$, a pink precipitate slowly appears at the bottom of the beaker. The mixture was stirred continuously for 6 hours for complete reaction. The solution was then filtered through Whatman filter paper. The precipitate was washed with 2 mL of 0.5 M HCl to get rid of the unreacted organic part and then with water. The pink precipitate was then dried in vacuum and used for further experiments.

Single crystals of the hybrid $(C_{25}H_{26}N_4)_4[SiW_{11}O_{39}]$ were isolated from DMSO-water mixture. For crystallization 3 mg of $(C_{25}H_{26}N_4)_4[SiW_{11}O_{39}]$ was dissolved in 2 mL of dimethyl sulfoxide and 2 mL of water was added to it. After one week red colored crystals were collected.

Preparation of the Soft-oxometalate (SOM) vesicle and Polymerization of the monomer

2 mg of $(C_{25}H_{26}N_4)_4[SiW_{11}O_{39}]$ was dissolved in 1 mL of DMSO at room temperature. 9 mL of water was then slowly added to the solution with constant stirring. White dispersion suggests the formation of soft-oxometalate vesicle in the dispersion. The dispersion was stored for more than 2 weeks. To change the vesicle size, the solvent ratio of water, DMSO was varied as 0.5:9.5, 1:9, 1.5:8.5, 2:8, and 2.5:7.5. Further experiments were carried out with these dispersions.

For polymerization, dispersion of the hybrid was kept in a vial inside a photoreactor (365 nm). After 24 hours, polymer is formed at the bottom of the vial. The solution turned blue-violet after keeping it for longer time. To change the chain length of the polymer, $K_8[SiW_{11}O_{39}]$ dissolved in water was added to a solution of tetrakis(4-aminophenyl)methane in DMSO. Amount of $K_8[SiW_{11}O_{39}]$ was varied to change the polymer chain length. These solutions were kept for 24 hours to polymerize. The polymers formed were separated from the solution and their chain lengths were measured by viscometry.

Single crystal X-ray crystallography

The red color crystal was diffracted on Supernova, Dual, Mo at zero, Eos diffractometer. During data collection the crystal was kept at 161K. Using Olex2, the structure was solved with the Superflip structure solution program using Charge flipping and refined with the ShelXL refinement package using Least Squares minimization. Single intensity data was collected with graphite-monochromatic Mo-K_α radiation. Among the 8683 unique reflections 5511 were considered to be observed with ($I>2\sigma(I)$). The final cycle of refinement, including the atomic coordinates, anisotropic thermal parameters, (W, Si atoms) and isotropic thermal parameters, converged at R=0.0541, R_w=0.1259. In the final difference map the highest peak was 1.702 and the deepest hole was -1.310. Unit cell parameters: a = 14.4300(6) Å; b = 31.5715(14) Å; c = 13.3320(6) Å. Space group: C2/m. The structure can be obtained by quoting the CCDC number 1580136.

Fourier Transform Infra-Red spectroscopy (FT-IR)

FT-IR spectra were recorded by Perkin Elmer spectrum Rx1 Spectrophotometer. In each case 1 mg of the sample was mixed with dry KBr and pellet was prepared from the mixture. The spectra were recorded in the range of 400-4000 cm^{-1}.

Scanning Electron Microscopy (SEM), Transmission Electron Microscopy (TEM) Energy-Dispersive X-ray spectroscopy (EDS)

A Carl Zeiss SUPRA55VP field emission scanning electron microscope was used for the SEM analysis. The samples were dried properly on silicon wafer pellets under ambient conditions. Samples were then coated with Au-Pt by vapor deposition before SEM measurement. The TEM and EDS measurements were accomplished with JEOL JEM-2100F transmission electron microscope operated at an accelerating voltage of 200 kV.

Electronic Absorption Spectroscopy (EAS)

All samples were properly diluted before the experiment. 3 mL of sample solution was taken in a quartz cuvette and EAS were recorded on a U-4100 spectrophotometer from 200 cm^{-1} to 1000 cm^{-1}.

Molecular weight measurement

Polymers of tetrakis(4-aminophenyl)methane were dissolved in conc. H_2SO_4. The solutions were sonicated and kept for 24 hours to make sure the polymer completely dissolved in the solution. The reduced viscosities of these samples were determined at 25°C in concentrated sulphuric acid using an Ubbelohde viscometer, average molecular weights were calculated using the Mark–Houwink relation-

$$[\boldsymbol{\eta}] = \boldsymbol{K}\overline{\boldsymbol{M}}_{\boldsymbol{v}}^{\boldsymbol{\alpha}}$$

Where, $[\boldsymbol{\eta}]$ = intrinsic viscosity, $\overline{\boldsymbol{M}}_{\boldsymbol{v}}$= Molecular weight and $\boldsymbol{K}$ and $\boldsymbol{\alpha}$ are Mark–Houwink constants.

The reduced viscosity of the polymers were calculated by measuring the viscometer drain times-

$$\boldsymbol{\eta_{red}} = (\boldsymbol{t} - \boldsymbol{t_0})/\boldsymbol{ct_0}$$

Where 't_0' was the viscometer drain time of sulfuric acid only, 't' was the viscometer drain time for a polymer solution of a known concentration (s), and 'c' was the known polymer concentration (g/dL). η_{red} was then plotted versus the polymer concentration for five concentrations, and a linear extrapolation to zero concentration produced the value of [η]. After putting the values of [η], K and α in the Mark–Houwink equation, the viscosity average molecular weight of the polymers were measured.

Dynamic light scattering (DLS)

A Malvern Zetaseizer instrument was used for both the DLS and zeta-potential measurements. A folded capillary cell was used for the analysis and an infinitely diluted dispersion (≈1 mL) was added to it. Note that the dilution was continued until a single beam of scattered laser was seen passing through the dispersion. DLS data provided the hydrodynamic radius (R_h) of the SOM vesicles as well as the particle-size distribution in the dispersion. The analysis chamber operated at ambient room temperature (25±0.1°C).

Electrochemical Measurements

All electrochemical measurements were performed using CHI400A instrument with a three-electrode cell configuration. Pt wire, glassy carbon electrode and Ag/AgCl electrodes were used as counter, working and reference electrode respectively. All the samples were dispersed in DMSO/H_2O and mixed with proper amount of Nafion solution (in ethanol). Glassy carbon electrode was properly polished each time before use. Thin films were prepared by drop casting the sample solution on the glassy carbon surface and drying overnight. All the measurements were done in 0.5 H_2SO_4 solution within the potential range of -0.2 V to 1 V.

N_2 Sorption measurement

Nitrogen sorption experiments were performed at 77 K up to 1 bar using a nanometric sorption analyser AutosorbiQ MP (Quantachrome Instruments). Samples were evacuated at 150° C for 12 h using a turbo molecular vacuum pump before analysis. Specific surface areas were calculated from nitrogen adsorption data by multipoint BET analysis. Pore size distributions were calculated from the N_2 adsorption isotherms using quenched solid density functional theory method which gives the least fitting error.

NMR Spectroscopy

^1H NMR and ^{13}C spectra were recorded at 500 MHz (^1H) and 125 MHZ (^{13}C) on a Bruker 500 MHz. Chemical shifts (δ) are reported in ppm, using the residual solvent peak in d^6-DMSO as internal standard.

General procedure for aniline oxidation

In a 10 mL reaction vial, 5 mg of polymer-SOM hybrid was dispersed in 5 mL of acetonitrile. 30% H_2O_2 (3 mmol) and 1 mmol of aniline was then added to the solution. The mixture was then stirred for 4 hours at 50ºC. Small aliquots of the reaction mixture were taken intermittently to check the progress of the reaction and analysed by gas chromatography. After completion water was added to the mixture to separate the catalyst from the reaction mixture. The catalyst was then filtered and washed with water several times. The recovered catalyst was then dried in vacuuo and reused for next cycle.

GC-MS analysis

After catalysis experiment, the reaction mixture was quenched with dilute HCl solution. The products were then extracted with ethyl acetate and used for GC-MS analysis. Each time a 0.5 µl solution was used for analysis. A Trace 1300 GC and ISQ qd single quadruple Mass spectrometer with a TG-5MS capillary column (30 m X 0.32 mm X 0.25µm) from Thermo Fisher Scientific was used for GC-MS analysis.

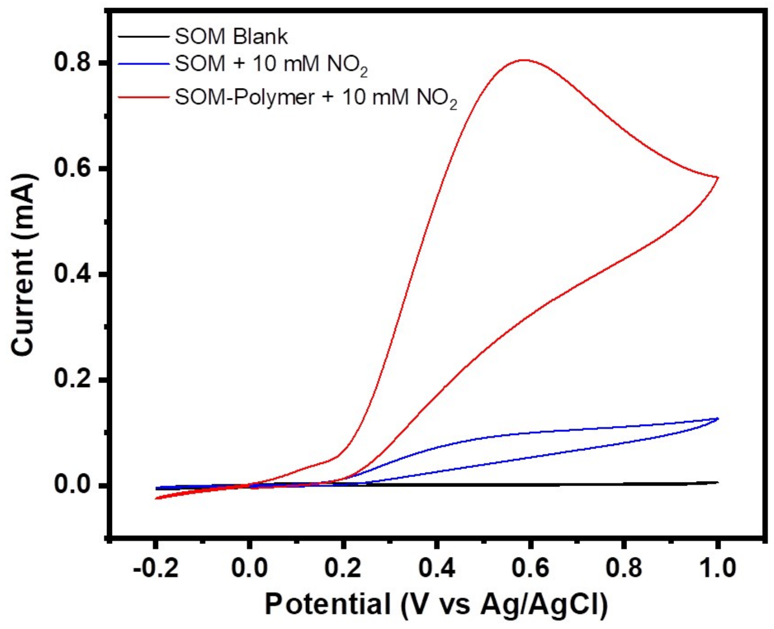

Figure S1: Comparison of nitrite oxidation between $(C_{25}H_{26}N_4)_4[SiW_{11}O_{39}]$ soft-oxometalate and its polymer hybrid.

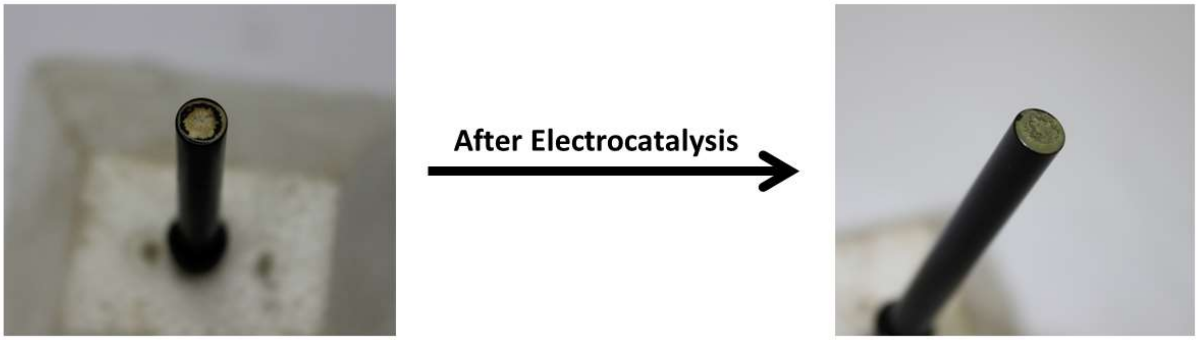

Figure S2: Colour change of the $(C_{25}H_{26}N_4)_4[SiW_{11}O_{39}]$ soft-oxometalate during electrocatalysis.

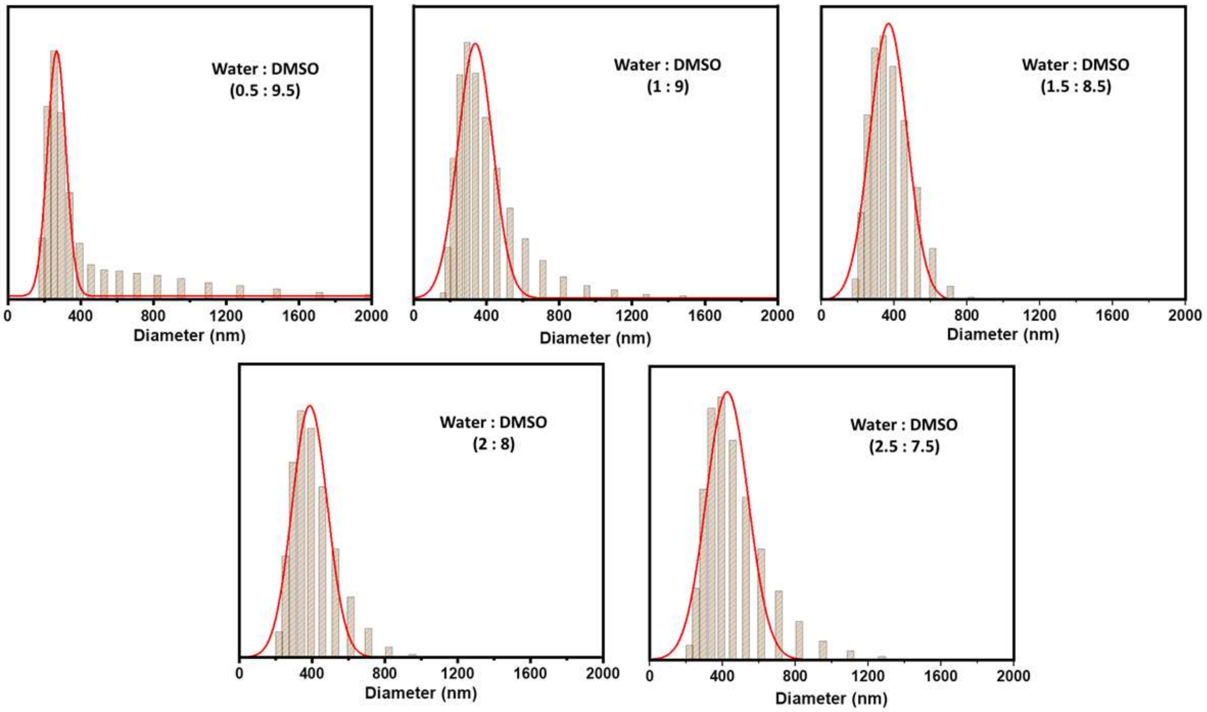

Figure S3: Size distribution of the $(C_{25}H_{26}N_4)_4[SiW_{11}O_{39}]$ soft-oxometalate vesicle in different DMSO : H_2O mixture determined by DLS.

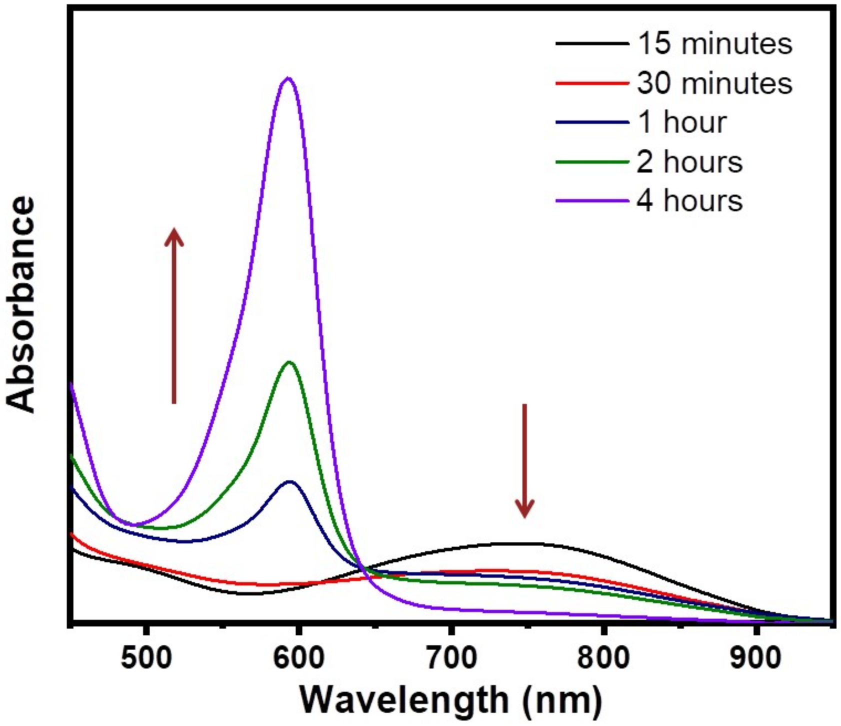

Figure S4: EAS of $(C_{25}H_{26}N_4)_4[SiW_{11}O_{39}]$ hybrid during polymerization. The peak at 744 nm arises because of the excitation of silicotungstate unit and the peak at 600 nm is due to the polymer of tetrakis(4-aminophenyl)methane.

Table S1: Oxidation of aniline by SOM-polymer in different solvents

Catalyst	Solvent	Conversion	Selectivity	Recovery
SOM-Polymer	H_2O	11%	75	95
	DMSO	92%	95	71
	Acetonitrile	96%	94	91
	DMSO:H_2O (9:1)	72%	81	79
	CH_2Cl_2	53%	95	93

Table S2: Molecular weight obtained from the value of [η] using the Mark–Houwink equation

Value of [η] (dL/g)	Molecular Weight (M_w)
0.851	14033
0.498	9465
0.409	8185
0.326	6926
0.040	1482

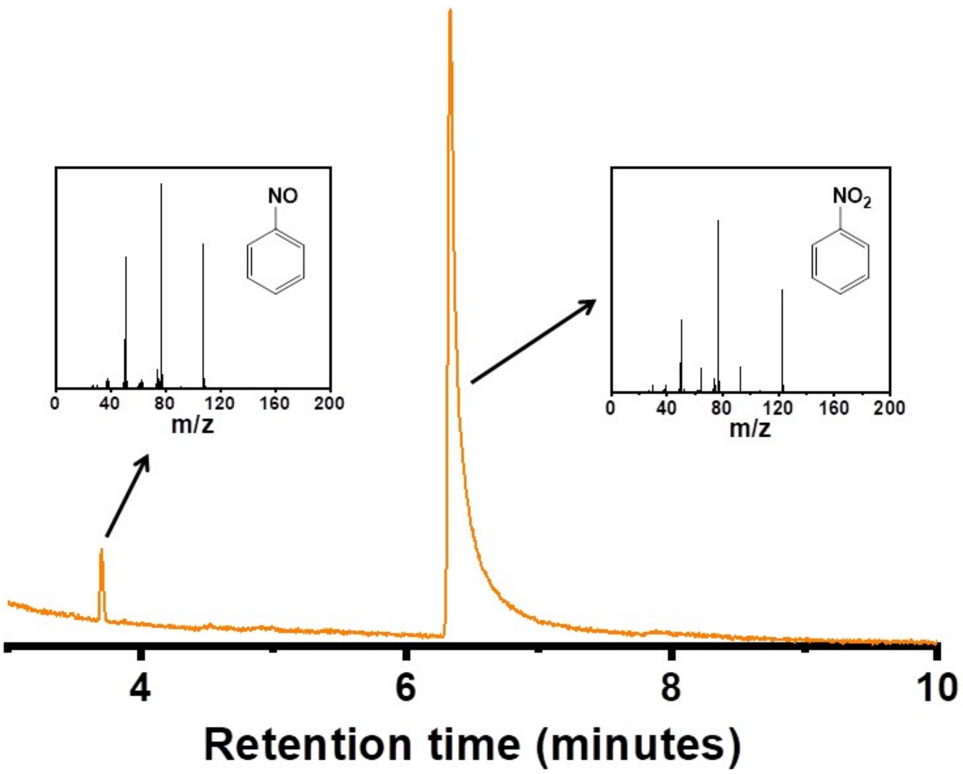

Figure S5: GC-MS spectrum of reaction mixture after aniline oxidation by SOM-polymer.

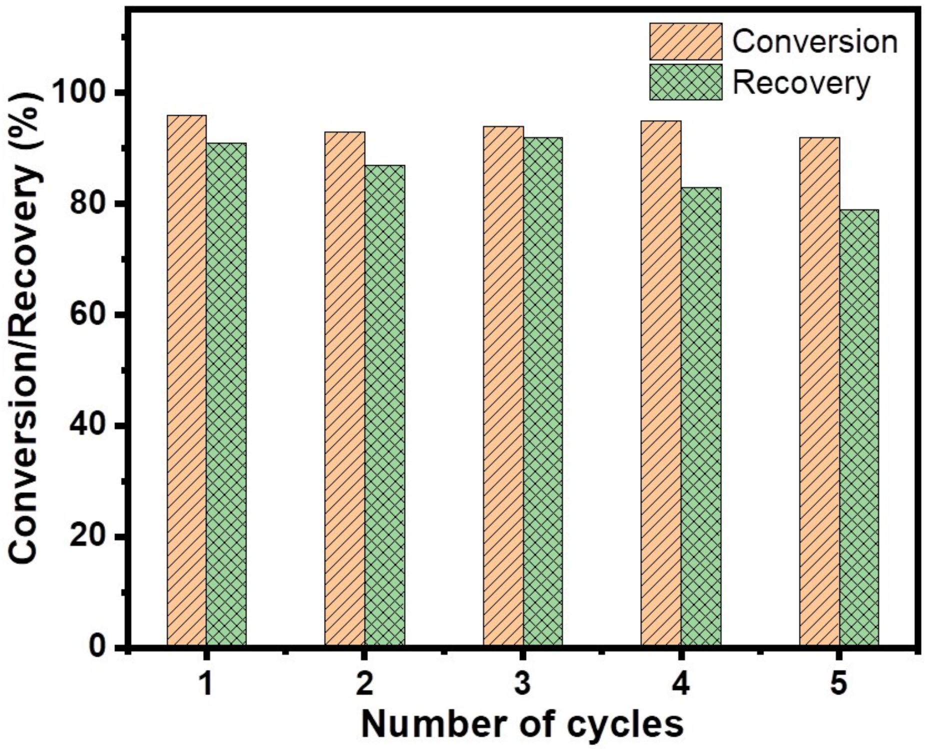

Figure S6: Conversion and recovery of SOM-polymer after aniline oxidation.

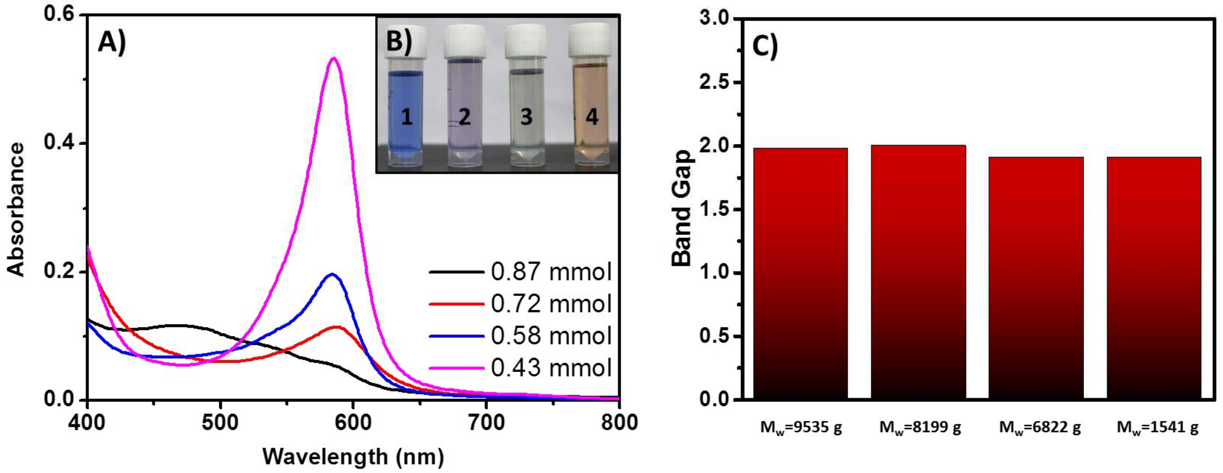

Figure S7: A) Electronic absorption spectra and B) Colours [1 → 4 lower to higher concentration] of the different chain length polymer in DMSO solution. C) Band gap energy of the different chain length polymer.

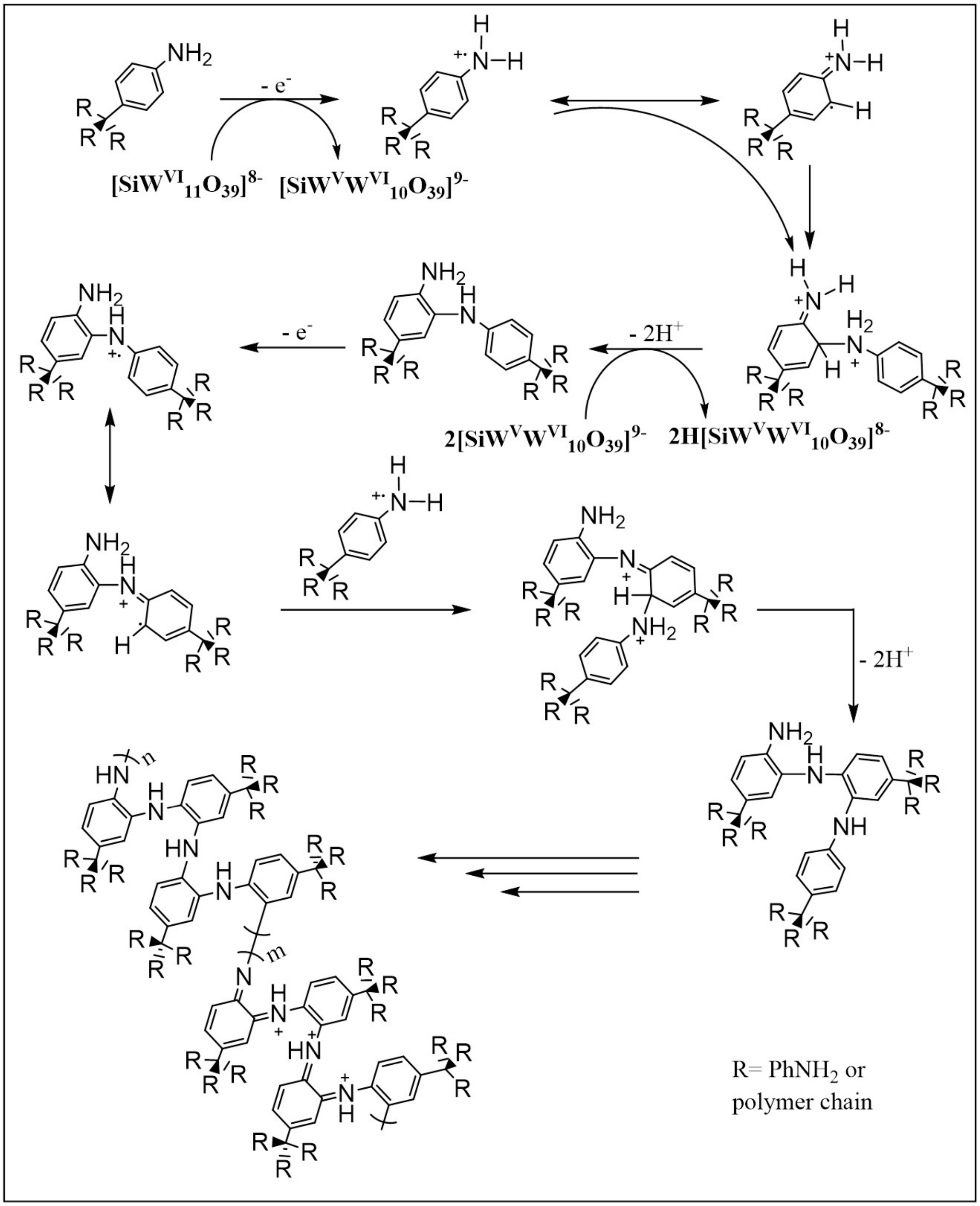

Figure S8: Proposed mechanism for the polymerization of tetrakis(4-aminophenyl)methane.

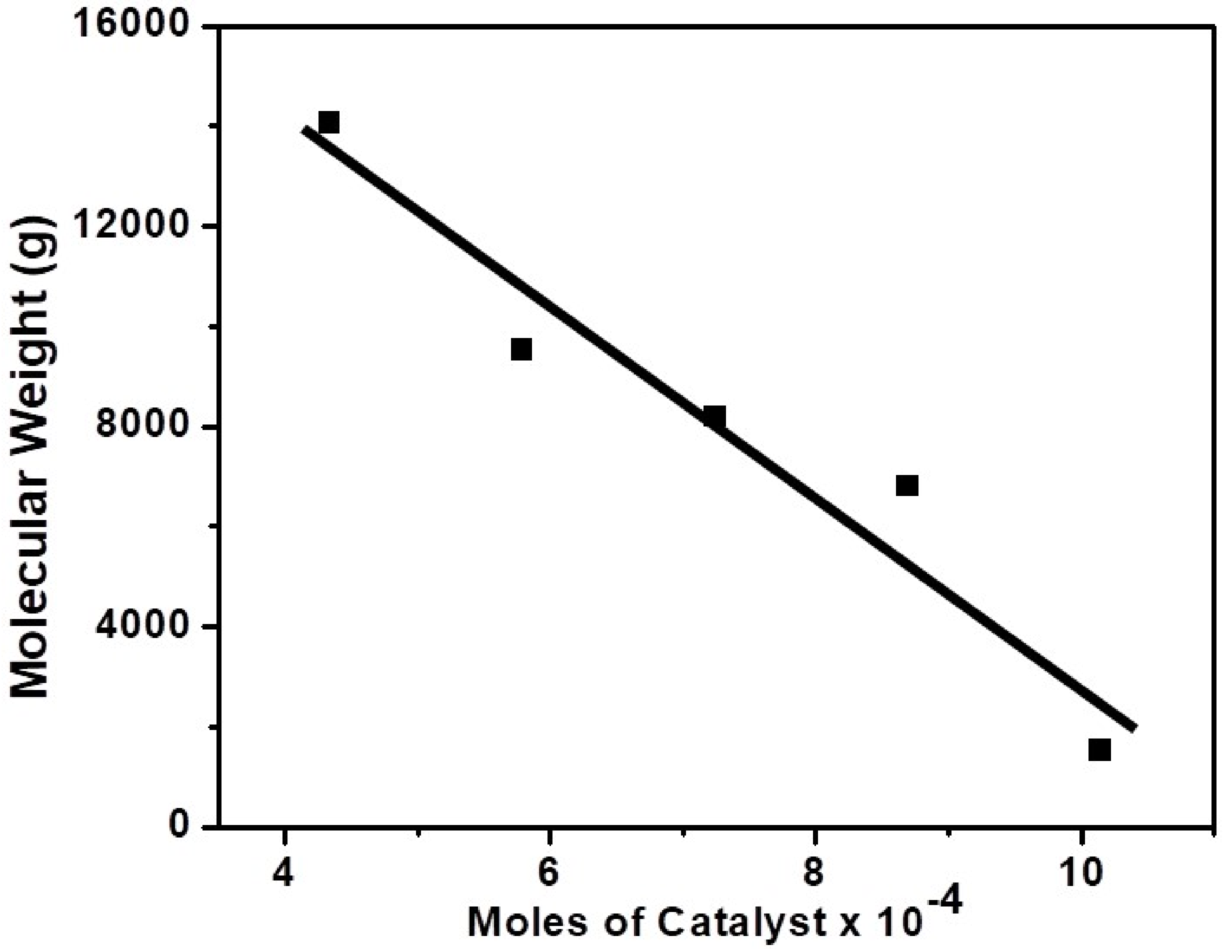

Figure S9: Plot of molecular weight of the polymer with respect to moles of oxometalate loading.

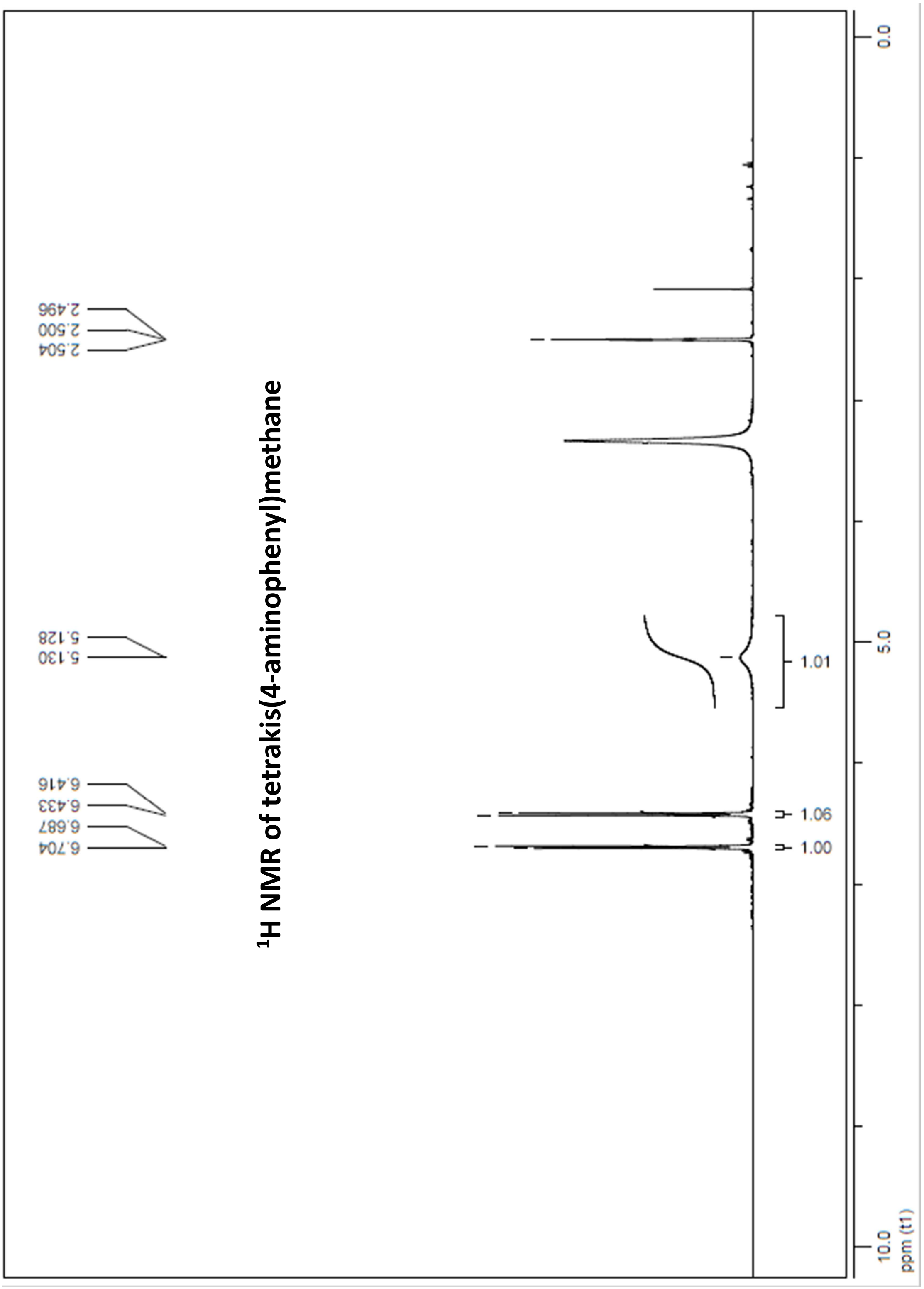

Figure S10: ^{1}H NMR of tetrakis(4-aminophenyl)methane

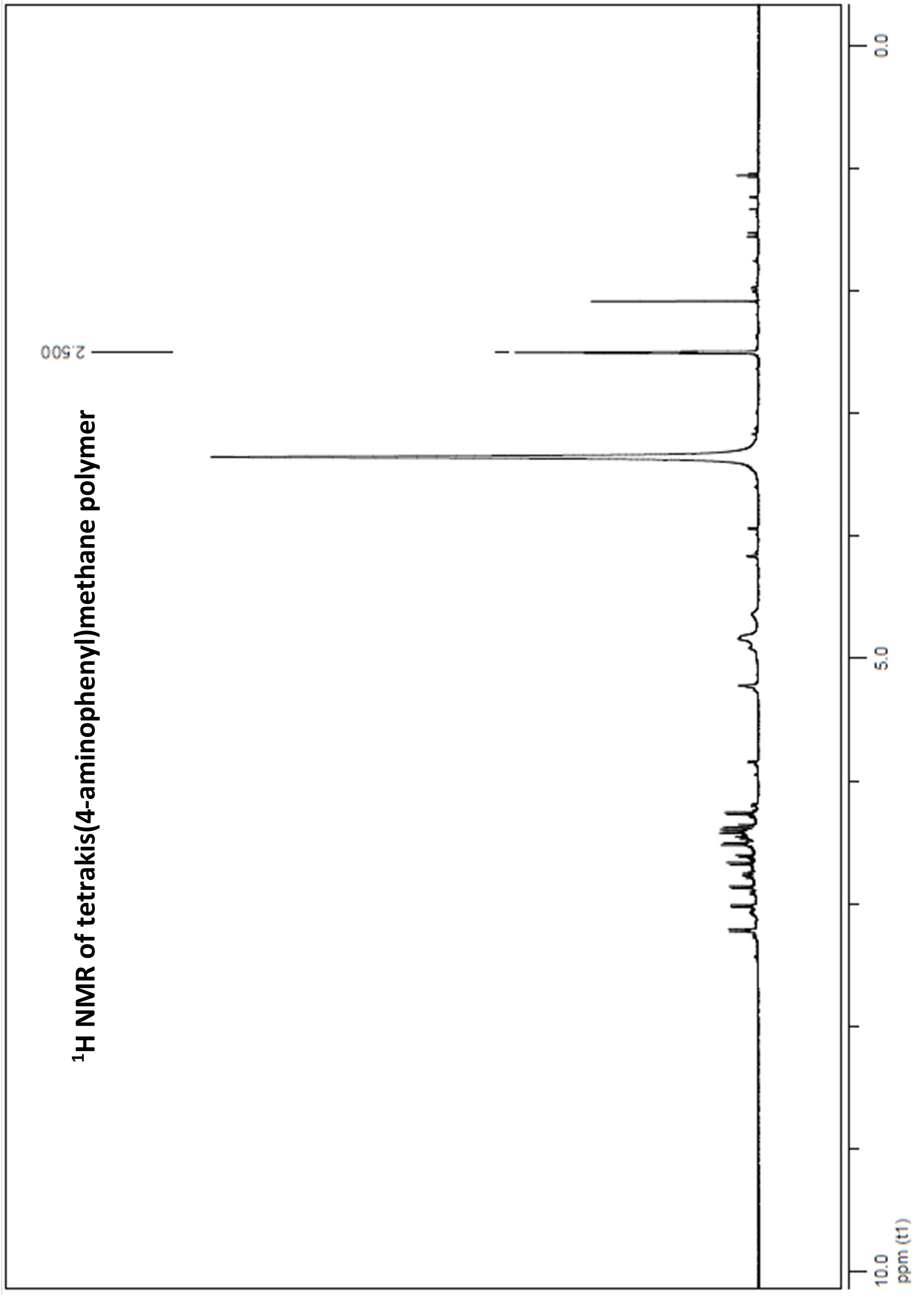

Figure 11: ^{1}H NMR of tetrakis(4-aminophenyl)methane polymer

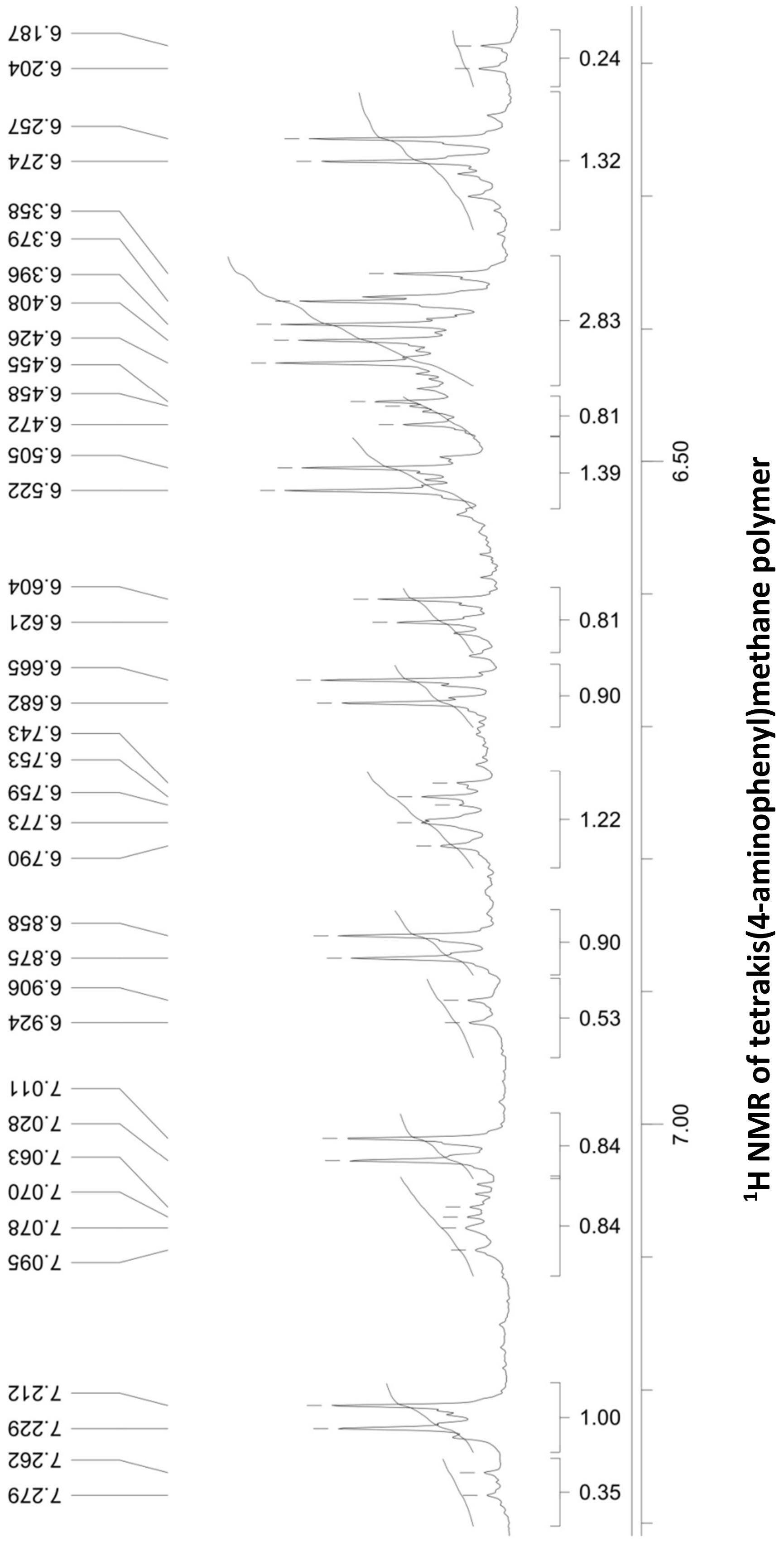

Figure S12: Expanded image of ^{1}H NMR of tetrakis(4-aminophenyl)methane polymer

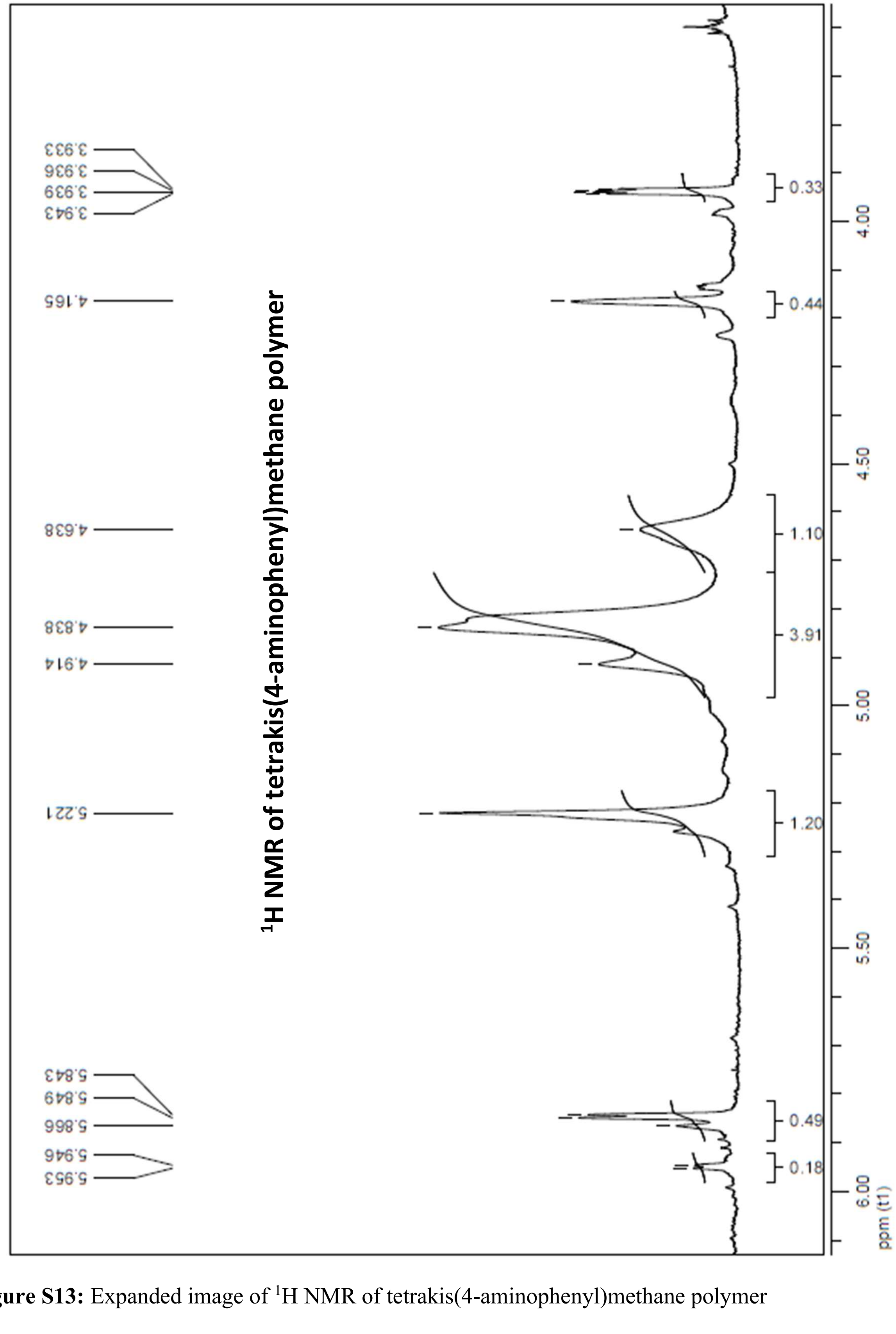

Figure S13: Expanded image of ^{1}H NMR of tetrakis(4-aminophenyl)methane polymer

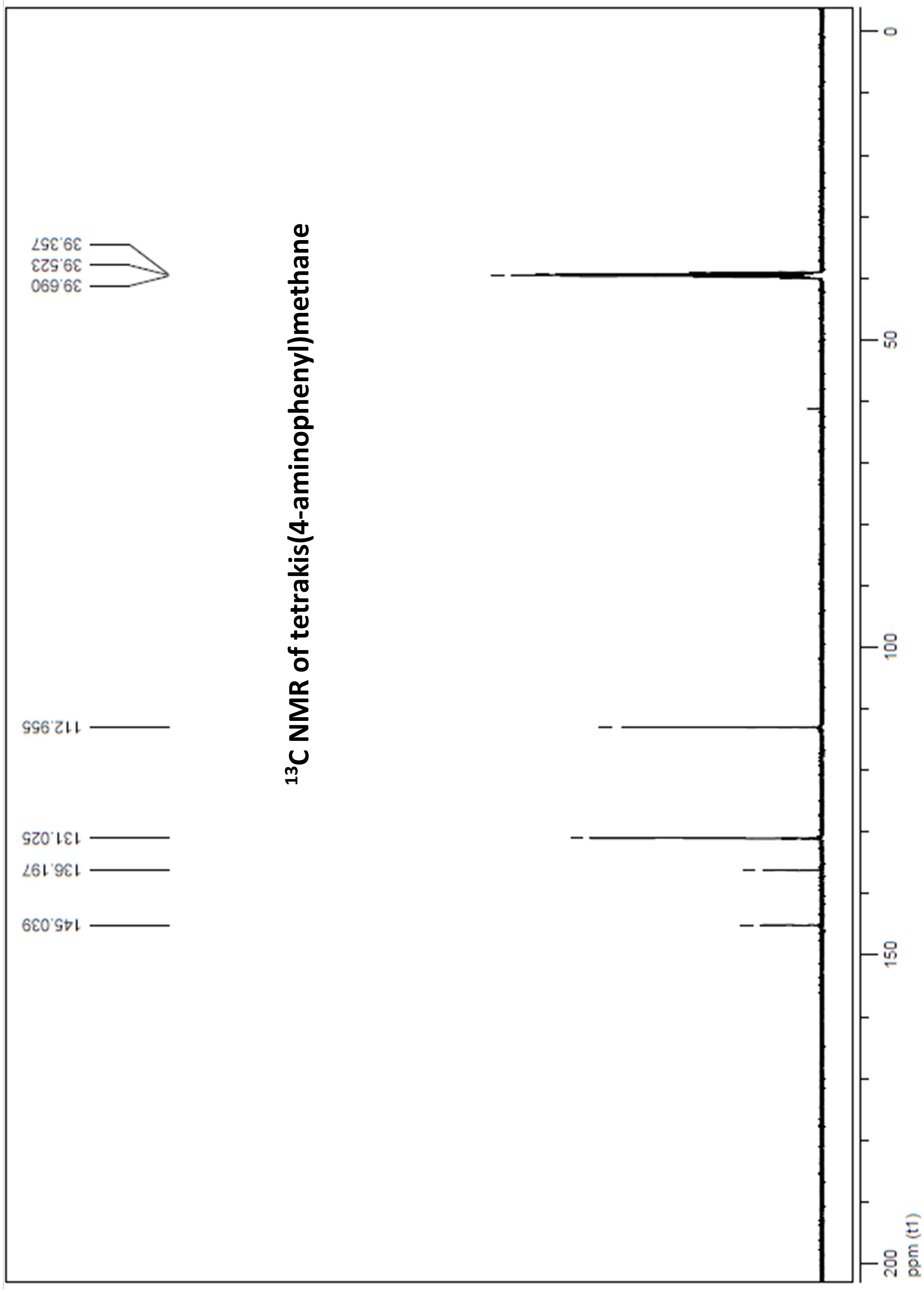

Figure S14: ^{13}C NMR of tetrakis(4-aminophenyl)methane

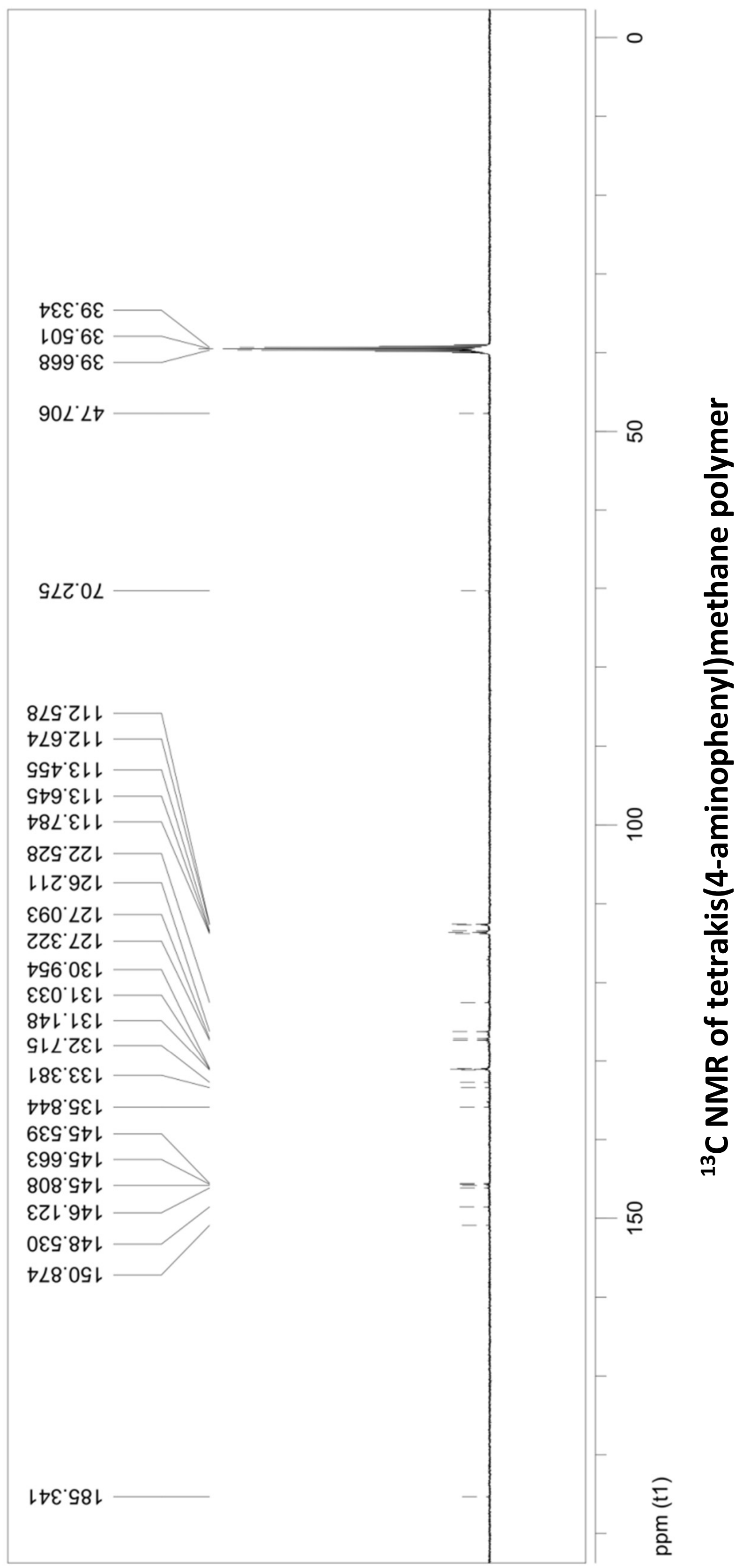

Figure S15: ^{13}C NMR of tetrakis(4-aminophenyl)methane polymer

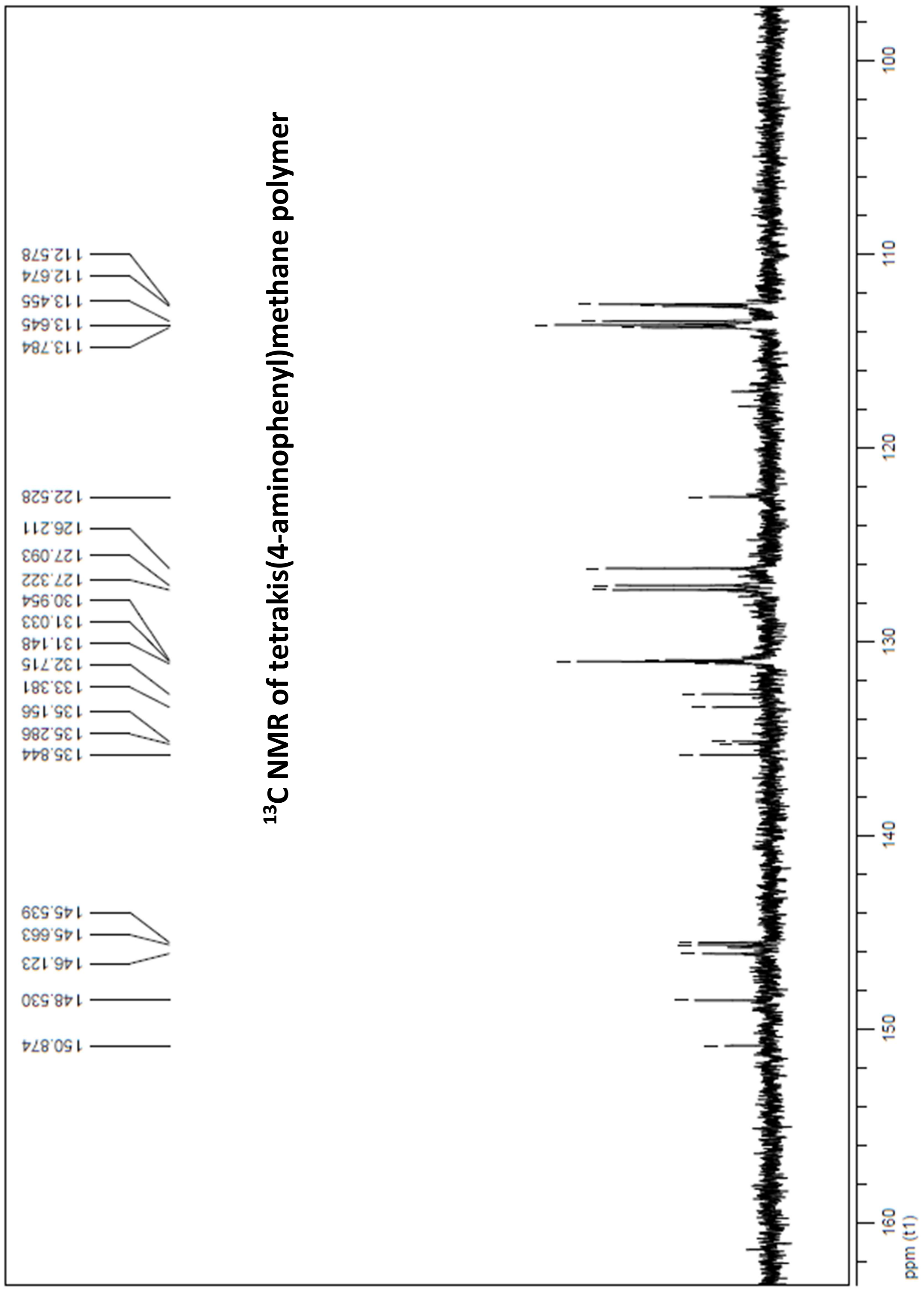

Figure S16: Expanded image of ^{13}C NMR of tetrakis(4-aminophenyl)methane polymer

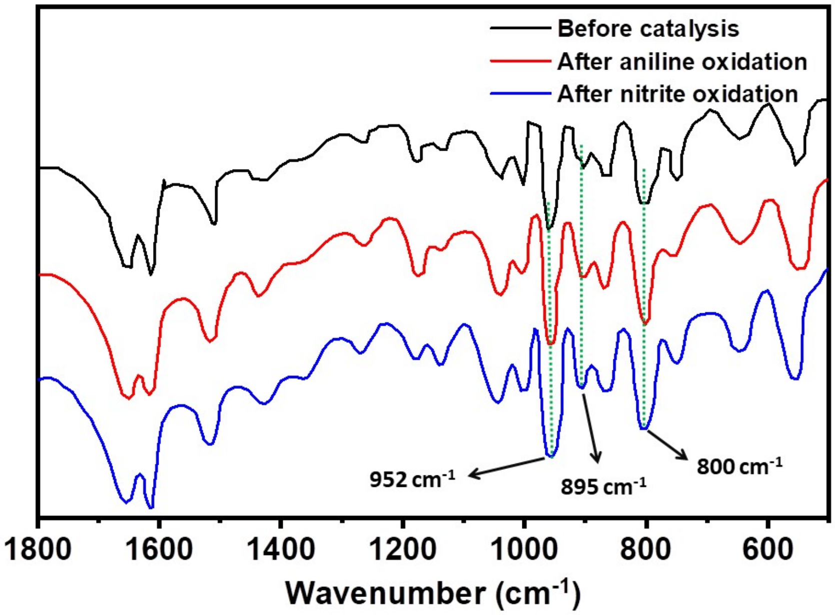

4

Figure S17: Comparison of IR spectra of the SOM-polymer hybrid- before catalysis, after aniline oxidation and after nitrite oxidation.

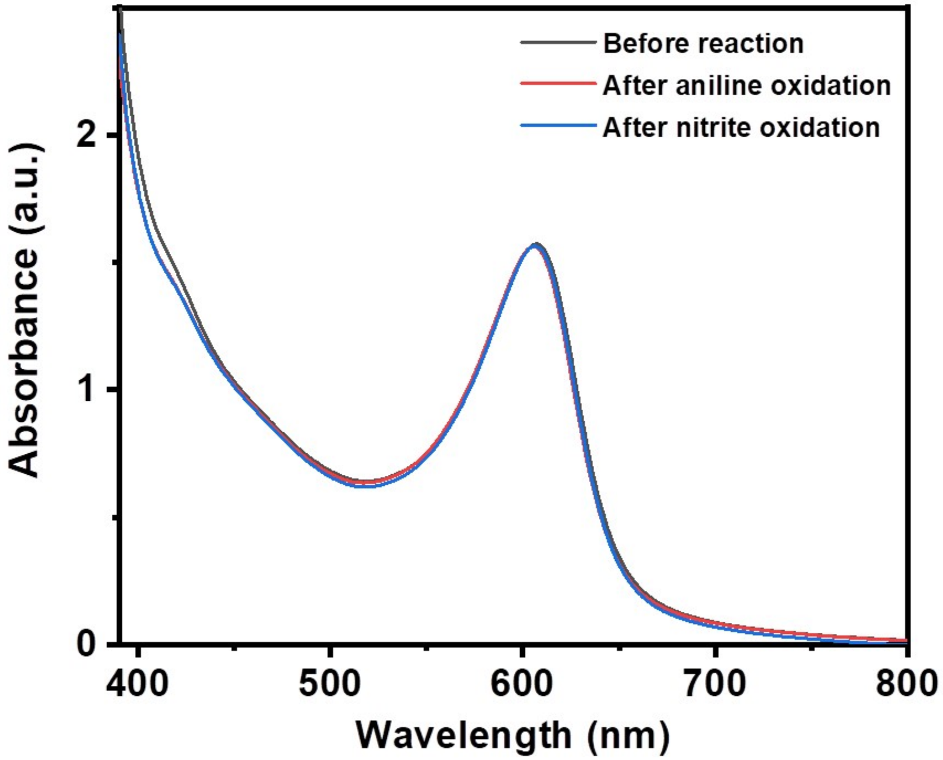

Figure S18: Comparison of EAS spectra of the SOM-polymer hybrid- before catalysis, after aniline oxidation and after nitrite oxidation.

CheckCIF/PLATON report

Structure factors have been supplied for datablock(s) l_siw-k

THIS REPORT IS FOR GUIDANCE ONLY. IF USED AS PART OF A REVIEW PROCEDURE FOR PUBLICATION, IT SHOULD NOT REPLACE THE EXPERTISE OF AN EXPERIENCED CRYSTALLOGRAPHIC REFEREE.

No syntax errors found. CIF dictionary Interpreting this report

Datablock:l_siw-k

Bond precision: C-C = 0.0175 A Wavelength=0.71073

Cell:	a=14.4300(6)	b=31.5715(14)	c=13.3320(6)
	alpha=90	beta=92.284(4)	gamma=90
Temperature:	161 K		

	Calculated	Reported
Volume	6068.9(5)	6068.9(5)
Space group	C 2/m	C 1 2/m 1
Hall group	-C 2y	-C 2y
Moiety formula	O29 W11, 4(C25 H26 N4), O4 Si, 6(O) [+ solvent]	O39 Si W11, 4(C25 H26 N4)
Sum formula	C100 H104 N16 O39 Si W11 [+ solvent]	C100 H100 N16 O39 Si W11
Dx,g cm-3	2.301	2.299
Z	2	2
Mu (mm-1)	10.470	10.470
F000	3912.1	3904.0
F000’	3896.75	
h,k,lmax	17,38,16	17,38,16
Nref	5672	5511
Tmin,Tmax	0.085,0.123	0.021,0.045
Tmin’	0.064	

Correction method= # Reported T Limits: Tmin=0.021 Tmax=0.045

AbsCorr = MULTI-SCAN

Data completeness= 0.972 Theta(max)= 25.350

R(reflections)= 0.0541(5009) wR2(reflections)= 0.1294(5511) S = 1.117 Npar= 419

The following ALERTS were generated. Each ALERT has the format

test-name_ALERT_alert-type_alert-level.

Click on the hyperlinks for more details of the test.

Alert level C

PLAT029_ALERT_3_C _diffrn_measured_fraction_theta_fullvalue Low . 0.976 Note PLAT234_ALERT_4_C Large Hirshfeld Difference W001 -- O007 .. 0.16 Ang. PLAT234_ALERT_4_C Large Hirshfeld Difference W001 -- O00L .. 0.19 Ang. PLAT234_ALERT_4_C Large Hirshfeld Difference W003 -- O00B .. 0.16 Ang. PLAT241_ALERT_2_C High 'MainMol' Ueq as Compared to Neighbors of O007 Check PLAT241_ALERT_2_C High 'MainMol' Ueq as Compared to Neighbors of O00A Check PLAT241_ALERT_2_C High 'MainMol' Ueq as Compared to Neighbors of O011 Check PLAT242_ALERT_2_C Low 'MainMol' Ueq as Compared to Neighbors of C00W Check PLAT242_ALERT_2_C Low 'MainMol' Ueq as Compared to Neighbors of C010 Check PLAT334_ALERT_2_C Small Average Benzene C-C Dist. C00J -C017 1.35 Ang. PLAT334_ALERT_2_C Small Average Benzene C-C Dist. C00Q -C00X 1.37 Ang. PLAT342_ALERT_3_C Low Bond Precision on C-C Bonds .... 0.01746 Ang. PLAT411_ALERT_2_C Short Inter H...H Contact H019 .. H019 . 2.05 Ang. PLAT417_ALERT_2_C Short Inter D-H..H-D H10C .. H11B .. 2.12 Ang. PLAT420_ALERT_2_C D-H Without Acceptor N8 -- H8A ... Please Check PLAT420_ALERT_2_C D-H Without Acceptor N8 -- H8B ... Please Check PLAT420_ALERT_2_C D-H Without Acceptor N10 -- H10A ... Please Check PLAT420_ALERT_2_C D-H Without Acceptor N10 -- H10C ... Please Check PLAT420_ALERT_2_C D-H Without Acceptor N11 -- H11B ... Please Check PLAT711_ALERT_1_C BOND Unknown or Inconsistent Label .... O

O01BA W001

PLAT711_ALERT_1_C BOND Unknown or Inconsistent Label .... O O00LA W001

PLAT711_ALERT_1_C BOND Unknown or Inconsistent Label .... O O00BA W001

PLAT711_ALERT_1_C BOND Unknown or Inconsistent Label .... O O01BA W001

PLAT711_ALERT_1_C BOND Unknown or Inconsistent Label .... O O015A W004

PLAT711_ALERT_1_C BOND Unknown or Inconsistent Label .... O O0B W004

PLAT711_ALERT_1_C BOND Unknown or Inconsistent Label .... O O00HA W004

PLAT711_ALERT_1_C BOND Unknown or Inconsistent Label .... O O00YA W004

PLAT711_ALERT_1_C BOND Unknown or Inconsistent Label .... O O00ZB W004

PLAT711_ALERT_1_C BOND Unknown or Inconsistent Label .... O O01AA O01A

PLAT712_ALERT_1_C ANGLE Unknown or Inconsistent Label .... O W001 O00LA W001

PLAT712_ALERT_1_C ANGLE Unknown or Inconsistent Label .... O W001 O01BA W001

PLAT712_ALERT_1_C ANGLE Unknown or Inconsistent Label .... O O01AA O01AA W001

PLAT712_ALERT_1_C ANGLE Unknown or Inconsistent Label .... O O01AA O01AA W002

PLAT712_ALERT_1_C ANGLE Unknown or Inconsistent Label .... O W001 O01BA W003

PLAT712_ALERT_1_C ANGLE Unknown or Inconsistent Label .... O W001 O01BA W003

PLAT712_ALERT_1_C ANGLE Unknown or Inconsistent Label .... O W001 O007A W004

PLAT712_ALERT_1_C ANGLE Unknown or Inconsistent Label .... O O01AA O01AA W004

PLAT712_ALERT_1_C ANGLE Unknown or Inconsistent Label .... O

W001 O01AA W004

PLAT712_ALERT_1_C ANGLE	Unknown or Inconsistent Label	O O00LA	W001	O00B
PLAT712_ALERT_1_C ANGLE	Unknown or Inconsistent Label	O O011A	W001	O00B
PLAT712_ALERT_1_C ANGLE	Unknown or Inconsistent Label	O O007A	W001	O00B
PLAT712_ALERT_1_C ANGLE	Unknown or Inconsistent Label	O O007A	W001	O00L
PLAT712_ALERT_1_C ANGLE	Unknown or Inconsistent Label	O O007A	W001	O011
PLAT712_ALERT_1_C ANGLE	Unknown or Inconsistent Label	O O00LA	W001	O011
PLAT712_ALERT_1_C ANGLE	Unknown or Inconsistent Label	O O011A	W001	O01A
PLAT712_ALERT_1_C ANGLE	Unknown or Inconsistent Label	O O00BA	W001	O01A
PLAT712_ALERT_1_C ANGLE	Unknown or Inconsistent Label	O O01BA	W001	O01A
PLAT712_ALERT_1_C ANGLE	Unknown or Inconsistent Label	O O007A	W001	O01A
PLAT712_ALERT_1_C ANGLE	Unknown or Inconsistent Label	O O00LA	W001	O01A
PLAT712_ALERT_1_C ANGLE	Unknown or Inconsistent Label	O O011A	W001	O01B
PLAT712_ALERT_1_C ANGLE	Unknown or Inconsistent Label	O O00LA	W001	O01B
PLAT712_ALERT_1_C ANGLE	Unknown or Inconsistent Label	O O00BA	W001	O01B
PLAT712_ALERT_1_C ANGLE	Unknown or Inconsistent Label	O O007A	W001	O01B
PLAT712_ALERT_1_C ANGLE	Unknown or Inconsistent Label	O O011A	W002	O00A
PLAT712_ALERT_1_C ANGLE	Unknown or Inconsistent Label	O O011A	W002	O00A
PLAT712_ALERT_1_C ANGLE	Unknown or Inconsistent Label	O O011A	W002	O00A
PLAT712_ALERT_1_C ANGLE	Unknown or Inconsistent Label	O O00AA	W002	O00A
PLAT712_ALERT_1_C ANGLE	Unknown or Inconsistent Label	O O011A	W002	O00A
PLAT712_ALERT_1_C ANGLE	Unknown or Inconsistent Label	O O011A	W002	O011
PLAT712_ALERT_1_C ANGLE	Unknown or Inconsistent Label	O O00AA	W002	O01A
PLAT712_ALERT_1_C ANGLE	Unknown or Inconsistent Label	O O011A	W002	O01A
PLAT712_ALERT_1_C ANGLE	Unknown or Inconsistent Label	O O011A	W002	O01A
PLAT712_ALERT_1_C ANGLE	Unknown or Inconsistent Label	O O011A	W002	O01A
PLAT712_ALERT_1_C ANGLE	Unknown or Inconsistent Label	O O01AA	W002	O01A
PLAT712_ALERT_1_C ANGLE	Unknown or Inconsistent Label	O O011A	W002	O01A
PLAT712_ALERT_1_C ANGLE	Unknown or Inconsistent Label	O O00AA	W002	O01A
PLAT712_ALERT_1_C ANGLE	Unknown or Inconsistent Label	O O00AA	W002	O01A
PLAT712_ALERT_1_C ANGLE	Unknown or Inconsistent Label	O O00AA	W002	O01A

PLAT712_ALERT_1_C ANGLE Unknown or Inconsistent Label .... O

O00BA W003 O00B

PLAT712_ALERT_1_C ANGLE Unknown or Inconsistent Label .... O O015A W003 O00H

PLAT712_ALERT_1_C ANGLE Unknown or Inconsistent Label .... O O00BA W003 O00H

PLAT712_ALERT_1_C ANGLE Unknown or Inconsistent Label .... O O00BA W003 O00H

PLAT712_ALERT_1_C ANGLE Unknown or Inconsistent Label .... O O015A W003 O00H

PLAT712_ALERT_1_C ANGLE Unknown or Inconsistent Label .... O O00BA W003 O015

PLAT712_ALERT_1_C ANGLE Unknown or Inconsistent Label .... O O00BA W003 O015

PLAT712_ALERT_1_C ANGLE Unknown or Inconsistent Label .... O O015A W003 O015

PLAT712_ALERT_1_C ANGLE Unknown or Inconsistent Label .... O O00BA W003 O015

PLAT712_ALERT_1_C ANGLE Unknown or Inconsistent Label .... O O00BA W003 O015

PLAT712_ALERT_1_C ANGLE Unknown or Inconsistent Label .... O O015A W003 O01B

PLAT712_ALERT_1_C ANGLE Unknown or Inconsistent Label .... O O00HA W003 O01B

PLAT712_ALERT_1_C ANGLE Unknown or Inconsistent Label .... O O00BA W003 O01B

PLAT712_ALERT_1_C ANGLE Unknown or Inconsistent Label .... O O00BA W003 O01B

PLAT712_ALERT_1_C ANGLE Unknown or Inconsistent Label .... O O015A W003 O01B

PLAT712_ALERT_1_C ANGLE Unknown or Inconsistent Label .... O O006B W003 O0

PLAT712_ALERT_1_C ANGLE Unknown or Inconsistent Label .... O O006B W003 O0

PLAT712_ALERT_1_C ANGLE Unknown or Inconsistent Label .... O O0BW003 O0

PLAT712_ALERT_1_C ANGLE Unknown or Inconsistent Label .... O O00AA W004 O007

PLAT712_ALERT_1_C ANGLE Unknown or Inconsistent Label .... O O00YA W004 O00H

PLAT712_ALERT_1_C ANGLE Unknown or Inconsistent Label .... O O007A W004 O00H

PLAT712_ALERT_1_C ANGLE Unknown or Inconsistent Label .... O O00AA W004 O00H

PLAT712_ALERT_1_C ANGLE Unknown or Inconsistent Label .... O O015A W004 O00H

PLAT712_ALERT_1_C ANGLE Unknown or Inconsistent Label .... O O007A W004 O00Y

PLAT712_ALERT_1_C ANGLE Unknown or Inconsistent Label .... O O00AA W004 O00Y

PLAT712_ALERT_1_C ANGLE Unknown or Inconsistent Label .... O O015A W004 O00Y

PLAT712_ALERT_1_C ANGLE Unknown or Inconsistent Label .... O O007A W004 O015

PLAT712_ALERT_1_C ANGLE Unknown or Inconsistent Label .... O O00AA W004 O015

PLAT712_ALERT_1_C ANGLE Unknown or Inconsistent Label .... O O00AA W004 O01A

PLAT712_ALERT_1_C ANGLE Unknown or Inconsistent Label .... O O007A W004 O01A

PLAT712_ALERT_1_C ANGLE Unknown or Inconsistent Label .... O O00HA W004 O01A

PLAT712_ALERT_1_C ANGLE Unknown or Inconsistent Label .... O

O015A W004 O01A

PLAT712_ALERT_1_C ANGLE Unknown or Inconsistent Label .... O O00YA W004 O01A

PLAT712_ALERT_1_C ANGLE Unknown or Inconsistent Label .... O O008B W004 O00Z

PLAT712_ALERT_1_C ANGLE Unknown or Inconsistent Label .... O O0BW004 O00Z

PLAT712_ALERT_1_C ANGLE Unknown or Inconsistent Label .... O O008B W004 O0

PLAT712_ALERT_1_C ANGLE Unknown or Inconsistent Label .... O O01BA SI05 O00H

PLAT712_ALERT_1_C ANGLE Unknown or Inconsistent Label .... O O01AA SI05 O00H

PLAT712_ALERT_1_C ANGLE Unknown or Inconsistent Label .... O O00HA SI05 O00H

PLAT712_ALERT_1_C ANGLE Unknown or Inconsistent Label .... O O01AA SI05 O00H

PLAT712_ALERT_1_C ANGLE Unknown or Inconsistent Label .... O O01AA SI05 O00H

PLAT712_ALERT_1_C ANGLE Unknown or Inconsistent Label .... O O01AA SI05 O00H

PLAT712_ALERT_1_C ANGLE Unknown or Inconsistent Label .... O O01AA SI05 O00H

PLAT712_ALERT_1_C ANGLE Unknown or Inconsistent Label .... O O01BA SI05 O00H

PLAT712_ALERT_1_C ANGLE Unknown or Inconsistent Label .... O O01BA SI05 O00H

PLAT712_ALERT_1_C ANGLE Unknown or Inconsistent Label .... O O01AA SI05 O00H

PLAT712_ALERT_1_C ANGLE Unknown or Inconsistent Label .... O O01BA SI05 O00H

PLAT712_ALERT_1_C ANGLE Unknown or Inconsistent Label .... O O01AA SI05 O00H

PLAT712_ALERT_1_C ANGLE Unknown or Inconsistent Label .... O O01AA SI05 O00H

PLAT712_ALERT_1_C ANGLE Unknown or Inconsistent Label .... O O01BA SI05 O01A

PLAT712_ALERT_1_C ANGLE Unknown or Inconsistent Label .... O O01BA SI05 O01A

PLAT712_ALERT_1_C ANGLE Unknown or Inconsistent Label .... O O01BA SI05 O01A

PLAT712_ALERT_1_C ANGLE Unknown or Inconsistent Label .... O O01AA SI05 O01A

PLAT712_ALERT_1_C ANGLE Unknown or Inconsistent Label .... O O01BA SI05 O01A

PLAT712_ALERT_1_C ANGLE Unknown or Inconsistent Label .... O O01AA SI05 O01A

PLAT712_ALERT_1_C ANGLE Unknown or Inconsistent Label .... O O01AA SI05 O01A

PLAT712_ALERT_1_C ANGLE Unknown or Inconsistent Label .... O O01BA SI05 O01A

PLAT712_ALERT_1_C ANGLE Unknown or Inconsistent Label .... O O01AA SI05 O01A

PLAT712_ALERT_1_C ANGLE Unknown or Inconsistent Label .... O O01BA SI05 O01A

PLAT712_ALERT_1_C ANGLE Unknown or Inconsistent Label .... O O01BA SI05 O01A

PLAT712_ALERT_1_C ANGLE Unknown or Inconsistent Label .... O O01AA SI05 O01A

PLAT712_ALERT_1_C ANGLE Unknown or Inconsistent Label .... O O01AA SI05 O01A

PLAT712_ALERT_1_C ANGLE Unknown or Inconsistent Label .... O

O01BA SI05 O01A

PLAT712_ALERT_1_C ANGLE Unknown or Inconsistent Label .... O O01BA SI05 O01B

PLAT712_ALERT_1_C ANGLE Unknown or Inconsistent Label .... O W002 O011A W001

PLAT712_ALERT_1_C ANGLE Unknown or Inconsistent Label .... O W002 O01AA W001

PLAT712_ALERT_1_C ANGLE Unknown or Inconsistent Label .... O W002 O01AA W004

PLAT712_ALERT_1_C ANGLE Unknown or Inconsistent Label .... O W003 O00BA W001

PLAT712_ALERT_1_C ANGLE Unknown or Inconsistent Label .... O W003 O015A W004

PLAT712_ALERT_1_C ANGLE Unknown or Inconsistent Label .... O W004 O00AA W002

PLAT712_ALERT_1_C ANGLE Unknown or Inconsistent Label .... O W004 O0B W003

PLAT712_ALERT_1_C ANGLE Unknown or Inconsistent Label .... O W004 O00HA W003

PLAT712_ALERT_1_C ANGLE Unknown or Inconsistent Label .... O W004 O00HA W003

PLAT712_ALERT_1_C ANGLE Unknown or Inconsistent Label .... O W004 O00HA W004

PLAT712_ALERT_1_C ANGLE Unknown or Inconsistent Label .... O W004 O00YA W004

PLAT712_ALERT_1_C ANGLE Unknown or Inconsistent Label .... O W004 O00ZB W004

PLAT712_ALERT_1_C ANGLE Unknown or Inconsistent Label .... O SI05O01BA W001

PLAT712_ALERT_1_C ANGLE Unknown or Inconsistent Label .... O SI05O01AA W001

PLAT712_ALERT_1_C ANGLE Unknown or Inconsistent Label .... O SI05O01BA W001

PLAT712_ALERT_1_C ANGLE Unknown or Inconsistent Label .... O SI05O01AA W002

PLAT712_ALERT_1_C ANGLE Unknown or Inconsistent Label .... O SI05O00HA W003

PLAT712_ALERT_1_C ANGLE Unknown or Inconsistent Label .... O SI05O01BA W003

PLAT712_ALERT_1_C ANGLE Unknown or Inconsistent Label .... O SI05O00HA W004

PLAT712_ALERT_1_C ANGLE Unknown or Inconsistent Label .... O SI05O01AA W004

PLAT712_ALERT_1_C ANGLE Unknown or Inconsistent Label .... O SI05O00HA W004

PLAT712_ALERT_1_C ANGLE Unknown or Inconsistent Label .... O SI05O01AA O01A

PLAT713_ALERT_1_C TORSION Unknown or Inconsistent Label .... O O00C W001 O00LA W001

PLAT713_ALERT_1_C TORSION Unknown or Inconsistent Label .... O O01AA W001 O00LA W001

PLAT713_ALERT_1_C TORSION Unknown or Inconsistent Label .... O O01BA W001 O00LA W001

PLAT713_ALERT_1_C TORSION Unknown or Inconsistent Label .... O O011A W001 O00LA W001

PLAT713_ALERT_1_C TORSION Unknown or Inconsistent Label .... O O00C W001 O011A W002

PLAT713_ALERT_1_C TORSION Unknown or Inconsistent Label .... O O00C W001 O007A W004

PLAT713_ALERT_1_C TORSION Unknown or Inconsistent Label .... O O00BA W001 O00LA W001

PLAT713_ALERT_1_C TORSION Unknown or Inconsistent Label .... O

O007A W001 O00LA W001

PLAT713_ALERT_1_C TORSION Unknown or Inconsistent Label .... O O01AA W001 O011A W002

PLAT713_ALERT_1_C TORSION Unknown or Inconsistent Label .... O O00BA W001 O011A W002

PLAT713_ALERT_1_C TORSION Unknown or Inconsistent Label .... O O01BA W001 O011A W002

PLAT713_ALERT_1_C TORSION Unknown or Inconsistent Label .... O O00LA W001 O011A W002

PLAT713_ALERT_1_C TORSION Unknown or Inconsistent Label .... O O007A W001 O011A W002

PLAT713_ALERT_1_C TORSION Unknown or Inconsistent Label .... O O00BA W001 O007A W004

PLAT713_ALERT_1_C TORSION Unknown or Inconsistent Label .... O O01AA W001 O007A W004

PLAT713_ALERT_1_C TORSION Unknown or Inconsistent Label .... O O00LA W001 O007A W004

PLAT713_ALERT_1_C TORSION Unknown or Inconsistent Label .... O O01BA W001 O007A W004

PLAT713_ALERT_1_C TORSION Unknown or Inconsistent Label .... O O011A W001 O007A W004

PLAT713_ALERT_1_C TORSION Unknown or Inconsistent Label .... O O00M W002 O011A W001

PLAT713_ALERT_1_C TORSION Unknown or Inconsistent Label .... O O01AA W002 O011A W001

PLAT713_ALERT_1_C TORSION Unknown or Inconsistent Label .... O O01AA W002 O011A W001

PLAT713_ALERT_1_C TORSION Unknown or Inconsistent Label .... O O011A W002 O011A W001

PLAT713_ALERT_1_C TORSION Unknown or Inconsistent Label .... O O00AA W002 O011A W001

PLAT713_ALERT_1_C TORSION Unknown or Inconsistent Label .... O O00AA W002 O011A W001

PLAT713_ALERT_1_C TORSION Unknown or Inconsistent Label .... O O00BA W003 O00BA W001

PLAT713_ALERT_1_C TORSION Unknown or Inconsistent Label .... O O00HA W003 O00BA W001

PLAT713_ALERT_1_C TORSION Unknown or Inconsistent Label .... O O01BA W003 O00BA W001

PLAT713_ALERT_1_C TORSION Unknown or Inconsistent Label .... O O015A W003 O00BA W001

PLAT713_ALERT_1_C TORSION Unknown or Inconsistent Label .... O O015A W003 O00BA W001

PLAT713_ALERT_1_C TORSION Unknown or Inconsistent Label .... O O006B W003 O0B W004

PLAT713_ALERT_1_C TORSION Unknown or Inconsistent Label .... O O0B W003 O0B W004

PLAT713_ALERT_1_C TORSION Unknown or Inconsistent Label .... O

PLAT713_ALERT_1_C TORSION Unknown or Inconsistent Label .... O O01AA W004 O00AA W002

PLAT713_ALERT_1_C TORSION Unknown or Inconsistent Label .... O O015A W004 O00AA W002

PLAT713_ALERT_1_C TORSION Unknown or Inconsistent Label .... O O00HA W004 O00AA W002

PLAT713_ALERT_1_C TORSION Unknown or Inconsistent Label .... O O007A W004 O00AA W002

PLAT713_ALERT_1_C TORSION Unknown or Inconsistent Label .... O O00YA W004 O00AA W002

PLAT713_ALERT_1_C TORSION Unknown or Inconsistent Label .... O O0B W004 O00ZB W004

PLAT713_ALERT_1_C TORSION Unknown or Inconsistent Label .... O O008B W004 O00ZB W004

PLAT713_ALERT_1_C TORSION Unknown or Inconsistent Label .... O

O01AA SI05 O01BA W001

PLAT713_ALERT_1_C TORSION Unknown or Inconsistent Label .... O O01AA SI05 O01BA W001

PLAT713_ALERT_1_C TORSION Unknown or Inconsistent Label .... O O00HA SI05 O01BA W001

PLAT713_ALERT_1_C TORSION Unknown or Inconsistent Label .... O O01AA SI05 O01BA W001

PLAT713_ALERT_1_C TORSION Unknown or Inconsistent Label .... O O01AA SI05 O01BA W001

PLAT713_ALERT_1_C TORSION Unknown or Inconsistent Label .... O O01BA SI05 O01AA O01AA

PLAT713_ALERT_1_C TORSION Unknown or Inconsistent Label .... O O00HA SI05 O01AA W001

PLAT713_ALERT_1_C TORSION Unknown or Inconsistent Label .... O O00HA SI05 O01BA W001

PLAT713_ALERT_1_C TORSION Unknown or Inconsistent Label .... O O01AA SI05 O01AA O01AA

PLAT713_ALERT_1_C TORSION Unknown or Inconsistent Label .... O O01AA SI05 O01BA W001

PLAT713_ALERT_1_C TORSION Unknown or Inconsistent Label .... O O00HA SI05 O01AA O01AA

PLAT713_ALERT_1_C TORSION Unknown or Inconsistent Label .... O O01AA SI05 O01BA W001

PLAT713_ALERT_1_C TORSION Unknown or Inconsistent Label .... O O00HA SI05 O01AA O01AA

PLAT713_ALERT_1_C TORSION Unknown or Inconsistent Label .... O O00HA SI05 O01BA W001

PLAT713_ALERT_1_C TORSION Unknown or Inconsistent Label .... O O01BA SI05 O01AA O01AA

PLAT713_ALERT_1_C TORSION Unknown or Inconsistent Label .... O O01AA SI05 O01BA W001

PLAT713_ALERT_1_C TORSION Unknown or Inconsistent Label .... O O01BA SI05 O01AA W001

PLAT713_ALERT_1_C TORSION Unknown or Inconsistent Label .... O O01AA SI05 O01BA W001

PLAT713_ALERT_1_C TORSION Unknown or Inconsistent Label .... O O01AA SI05 O01AA W001

PLAT713_ALERT_1_C TORSION Unknown or Inconsistent Label .... O O00HA SI05 O01BA W001

PLAT713_ALERT_1_C TORSION Unknown or Inconsistent Label .... O O01AA SI05 O01AA W001

PLAT713_ALERT_1_C TORSION Unknown or Inconsistent Label .... O O00HA SI05 O01AA W001

PLAT713_ALERT_1_C TORSION Unknown or Inconsistent Label .... O O01BA SI05 O01AA W001

PLAT713_ALERT_1_C TORSION Unknown or Inconsistent Label .... O O01BA SI05 O01AA W002

PLAT713_ALERT_1_C TORSION Unknown or Inconsistent Label .... O

PLAT713_ALERT_1_C TORSION Unknown or Inconsistent Label .... O O01BA SI05 O01AA W002

PLAT713_ALERT_1_C TORSION Unknown or Inconsistent Label .... O O00HA SI05 O01AA W002

PLAT713_ALERT_1_C TORSION Unknown or Inconsistent Label .... O O01AA SI05 O01AA W002

PLAT713_ALERT_1_C TORSION Unknown or Inconsistent Label .... O O01AA SI05 O01AA W002

PLAT713_ALERT_1_C TORSION Unknown or Inconsistent Label .... O O00HA SI05 O01AA W002

PLAT713_ALERT_1_C TORSION Unknown or Inconsistent Label .... O O01AA SI05 O01BA W003

PLAT713_ALERT_1_C TORSION Unknown or Inconsistent Label .... O O01AA SI05 O00HA W003

PLAT713_ALERT_1_C TORSION Unknown or Inconsistent Label .... O

O00HA SI05 O01BA W003

PLAT713_ALERT_1_C TORSION Unknown or Inconsistent Label .... O O01AA SI05 O01BA W003

PLAT713_ALERT_1_C TORSION Unknown or Inconsistent Label .... O O01BA SI05 O00HA W003

PLAT713_ALERT_1_C TORSION Unknown or Inconsistent Label .... O O01AA SI05 O00HA W003

PLAT713_ALERT_1_C TORSION Unknown or Inconsistent Label .... O O01AA SI05 O01BA W003

PLAT713_ALERT_1_C TORSION Unknown or Inconsistent Label .... O O01BA SI05 O00HA W003

PLAT713_ALERT_1_C TORSION Unknown or Inconsistent Label .... O O01AA SI05 O01BA W003

PLAT713_ALERT_1_C TORSION Unknown or Inconsistent Label .... O O00HA SI05 O01BA W003

PLAT713_ALERT_1_C TORSION Unknown or Inconsistent Label .... O O01AA SI05 O00HA W003

PLAT713_ALERT_1_C TORSION Unknown or Inconsistent Label .... O O01AA SI05 O00HA W003

PLAT713_ALERT_1_C TORSION Unknown or Inconsistent Label .... O O01AA SI05 O00HA W004

PLAT713_ALERT_1_C TORSION Unknown or Inconsistent Label .... O O01BA SI05 O01AA W004

PLAT713_ALERT_1_C TORSION Unknown or Inconsistent Label .... O O00HA SI05 O01AA W004

PLAT713_ALERT_1_C TORSION Unknown or Inconsistent Label .... O O01BA SI05 O00HA W004

PLAT713_ALERT_1_C TORSION Unknown or Inconsistent Label .... O O01AA SI05 O01AA W004

PLAT713_ALERT_1_C TORSION Unknown or Inconsistent Label .... O O01BA SI05 O00HA W004

PLAT713_ALERT_1_C TORSION Unknown or Inconsistent Label .... O O01AA SI05 O00HA W004

PLAT713_ALERT_1_C TORSION Unknown or Inconsistent Label .... O O01AA SI05 O00HA W004

PLAT713_ALERT_1_C TORSION Unknown or Inconsistent Label .... O O01BA SI05 O00HA W004

PLAT713_ALERT_1_C TORSION Unknown or Inconsistent Label .... O O01AA SI05 O00HA W004

PLAT713_ALERT_1_C TORSION Unknown or Inconsistent Label .... O O01AA SI05 O00HA W004

PLAT713_ALERT_1_C TORSION Unknown or Inconsistent Label .... O O01AA SI05 O00HA W004

PLAT713_ALERT_1_C TORSION Unknown or Inconsistent Label .... O O00HA SI05 O01AA W004

PLAT713_ALERT_1_C TORSION Unknown or Inconsistent Label .... O O01AA SI05 O00HA W004

PLAT713_ALERT_1_C TORSION Unknown or Inconsistent Label .... O

PLAT713_ALERT_1_C TORSION Unknown or Inconsistent Label .... O O01AA SI05 O00HA W004

PLAT713_ALERT_1_C TORSION Unknown or Inconsistent Label .... O O01BA SI05 O00HA W004

PLAT713_ALERT_1_C TORSION Unknown or Inconsistent Label .... O O01BA SI05 O01AA W004

PLAT713_ALERT_1_C TORSION Unknown or Inconsistent Label .... O O01AA SI05 O01AA W004

PLAT906_ALERT_3_C Large K value in the Analysis of Variance ...... 3.334 Check PLAT910_ALERT_3_C Missing # of FCF Reflection(s) Below Theta(Min). 8 Note PLAT911_ALERT_3_C Missing # FCF Refl Between THmin & STh/L= 0.600 125 Report PLAT975_ALERT_2_C Check Calcd Residual Density 0.57A From O00L 1.15 eA-3

PLAT976_ALERT_2_C Check Calcd Residual Density 0.72A From O00L -1.04 eA-3

PLAT976_ALERT_2_C Check Calcd Residual Density 0.60A From O01A -0.67 eA-3

PLAT976_ALERT_2_C Check Calcd Residual Density 1.04A From N11 -0.66 eA-3
PLAT977_ALERT_2_C Check the Negative Difference Density on H9A -0.36 eA-3
PLAT978_ALERT_2_C Number C-C Bonds with Positive Residual Density. 0 Info

---

Alert level G

FORMU01_ALERT_1_G There is a discrepancy between the atom counts in the

_chemical_formula_sum and _chemical_formula_moiety. This is usually due to the moiety formula being in the wrong format.

Atom count from _chemical_formula_sum: C100 H100 N16 O39 Si1 W11

Atom count from _chemical_formula_moiety:C100 H104 N16 O39 Si1 W11

FORMU01_ALERT_2_G There is a discrepancy between the atom counts in the

_chemical_formula_sum and the formula from the _atom_site* data. Atom count from _chemical_formula_sum:C100 H100 N16 O39 Si1 W11

Atom count from the _atom_site data: C100 H104 N16 O39. Si1 W11. CELLZ01_ALERT_1_G Difference between formula and atom_site contents detected. CELLZ01_ALERT_1_G ALERT: Large difference may be due to a

symmetry error - see SYMMG tests

From the CIF: _cell_formula_units_Z 2

From the CIF: _chemical_formula_sum C100 H100 N16 O39 Si W11

TEST: Compare cell contents of formula and atom_site data

atom	Z*formula	cif sites	diff
C	200.00	200.00	0.00
H	200.00	208.00	-8.00
N	32.00	32.00	0.00
O	78.00	78.00	0.00
Si	2.00	2.00	0.00
W	22.00	22.00	0.00

PLAT003_ALERT_2_G Number of Uiso or Uij Restrained non-H Atoms ... 12 Report PLAT007_ALERT_5_G Number of Unrefined Donor-H Atoms .... 10 Report PLAT012_ALERT_1_G No _shelx_res_checksum found in CIF .... Please Check PLAT014_ALERT_1_G No _shelx_fab_checksum found in CIF .... Please Check PLAT041_ALERT_1_G Calc. and Reported SumFormula Strings Differ Please Check PLAT042_ALERT_1_G Calc. and Reported MoietyFormula Strings Differ Please Check PLAT068_ALERT_1_G Reported F000 Differs from Calcd (or Missing)... Please Check PLAT083_ALERT_2_G SHELXL Second Parameter in WGHT Unusually Large 134.21 Why ? PLAT186_ALERT_4_G The CIF-Embedded .res File Contains ISOR Records 1 Report PLAT187_ALERT_4_G The CIF-Embedded .res File Contains RIGU Records 1 Report PLAT300_ALERT_4_G Atom Site Occupancy of W001 is Constrained at 0.9167 Check PLAT300_ALERT_4_G Atom Site Occupancy of W002 is Constrained at 0.9167 Check PLAT300_ALERT_4_G Atom Site Occupancy of W003 is Constrained at 0.9167 Check PLAT300_ALERT_4_G Atom Site Occupancy of W004 is Constrained at 0.9167 Check PLAT300_ALERT_4_G Atom Site Occupancy of O006 is Constrained at 0.9167 Check PLAT300_ALERT_4_G Atom Site Occupancy of O008 is Constrained at 0.9167 Check PLAT300_ALERT_4_G Atom Site Occupancy of O00C is Constrained at 0.9167 Check PLAT300_ALERT_4_G Atom Site Occupancy of O00M is Constrained at 0.9167 Check PLAT300_ALERT_4_G Atom Site Occupancy of O00H is Constrained at 0.5 Check PLAT300_ALERT_4_G Atom Site Occupancy of O01A is Constrained at 0.5 Check PLAT300_ALERT_4_G Atom Site Occupancy of O01B is Constrained at 0.5 Check PLAT300_ALERT_4_G Atom Site Occupancy of O00Y is Constrained at 0.5 Check PLAT300_ALERT_4_G Atom Site Occupancy of O015 is Constrained at 0.5 Check PLAT300_ALERT_4_G Atom Site Occupancy of O00Z is Constrained at 0.5 Check PLAT300_ALERT_4_G Atom Site Occupancy of O0 is Constrained at 0.5 Check PLAT301_ALERT_3_G Main Residue Disorder ....(Resd 1).. 55% Note

PLAT302_ALERT_4_G Anion/Solvent/Minor-Residue Disorder (Resd 3).. 80% Note PLAT302_ALERT_4_G Anion/Solvent/Minor-Residue Disorder (Resd 4).. 100% Note PLAT302_ALERT_4_G Anion/Solvent/Minor-Residue Disorder (Resd 5).. 100% Note PLAT302_ALERT_4_G Anion/Solvent/Minor-Residue Disorder (Resd 6).. 100% Note PLAT302_ALERT_4_G Anion/Solvent/Minor-Residue Disorder (Resd 7).. 100% Note PLAT304_ALERT_4_G Non-Integer Number of Atoms (0.25) in Resd. # 4 Check

PLAT304_ALERT_4_G Non-Integer Number of Atoms (0.50) in Resd. # 5 Check PLAT304_ALERT_4_G Non-Integer Number of Atoms (0.25) in Resd. # 6 Check PLAT304_ALERT_4_G Non-Integer Number of Atoms (0.50) in Resd. # 7 Check PLAT311_ALERT_2_G Isolated Disordered Oxygen Atom (No H’s ?) ..... O00Y Check PLAT311_ALERT_2_G Isolated Disordered Oxygen Atom (No H’s ?) ..... O015 Check PLAT311_ALERT_2_G Isolated Disordered Oxygen Atom (No H’s ?) ..... O00Z Check PLAT311_ALERT_2_G Isolated Disordered Oxygen Atom (No H’s ?) ..... O0 Check PLAT432_ALERT_2_G Short Inter X...Y Contact Si05 .. O0 .. 3.24 Ang. PLAT432_ALERT_2_G Short Inter X...Y Contact Si05 .. O0 .. 3.24 Ang. PLAT432_ALERT_2_G Short Inter X...Y Contact Si05 .. O0 .. 3.24 Ang. PLAT432_ALERT_2_G Short Inter X...Y Contact Si05 .. O0 .. 3.24 Ang. PLAT432_ALERT_2_G Short Inter X...Y Contact Si05 .. O00Z .. 3.26 Ang. PLAT432_ALERT_2_G Short Inter X...Y Contact Si05 .. O00Z .. 3.26 Ang.

PLAT605_ALERT_4_G	Largest Solvent Accessible VOID in the Structure	352	A**3
PLAT720_ALERT_4_G	Number of Unusual/Non-Standard Labels	62	Note
PLAT764_ALERT_4_G	Overcomplete CIF Bond List Detected (Rep/Expd) .	1.29	Ratio
PLAT780_ALERT_1_G	Coordinates do not Form a Properly Connected Set	Please	Do !
PLAT860_ALERT_3_G	Number of Least-Squares Restraints	54	Note
PLAT869_ALERT_4_G	ALERTS Related to the use of SQUEEZE Suppressed	!	Info
PLAT912_ALERT_4_G	Missing # of FCF Reflections Above STh/L= 0.600	28	Note
PLAT933_ALERT_2_G	Number of OMIT Records in Embedded .res File ...	40	Note

---

0 **ALERT level A** = Most likely a serious problem - resolve or explain

0 **ALERT level B** = A potentially serious problem, consider carefully

260 **ALERT level C** = Check. Ensure it is not caused by an omission or oversight

57 **ALERT level G** = General information/check it is not something unexpected

241 ALERT type 1 CIF construction/syntax error, inconsistent or missing data

34 ALERT type 2 Indicator that the structure model may be wrong or deficient

7 ALERT type 3 Indicator that the structure quality may be low

34 ALERT type 4 Improvement, methodology, query or suggestion

1 ALERT type 5 Informative message, check

It is advisable to attempt to resolve as many as possible of the alerts in all categories. Often the minor alerts point to easily fixed oversights, errors and omissions in your CIF or refinement strategy, so attention to these fine details can be worthwhile. In order to resolve some of the more serious problems it may be necessary to carry out additional measurements or structure refinements. However, the purpose of your study may justify the reported deviations and the more

serious of these should normally be commented upon in the discussion or experimental section of a paper or in the "special_details" fields of the CIF. checkCIF was carefully designed to identify outliers and unusual parameters, but every test has its limitations and alerts that are not important

in a particular case may appear. Conversely, the absence of alerts does not guarantee there are no aspects of the results needing attention. It is up to the individual to critically assess their own results and, if necessary, seek expert advice.

Publication of your CIF in IUCr journals

A basic structural check has been run on your CIF. These basic checks will be run on all CIFs submitted for publication in IUCr journals (*Acta Crystallographica*, *Journal of Applied Crystallography*, *Journal of Synchrotron Radiation*); however, if you intend to submit to *Acta Crystallographica Section C* or *E* or *IUCrData*, you should make sure that full publication checks are run on the final version of your CIF prior to submission.

Publication of your CIF in other journals

Please refer to the *Notes for Authors* of the relevant journal for any special instructions relating to

CIF submission.

PLATON version of 13/08/2017; check.def file version of 27/07/2017

Datablock l_siw-k - ellipsoid plot

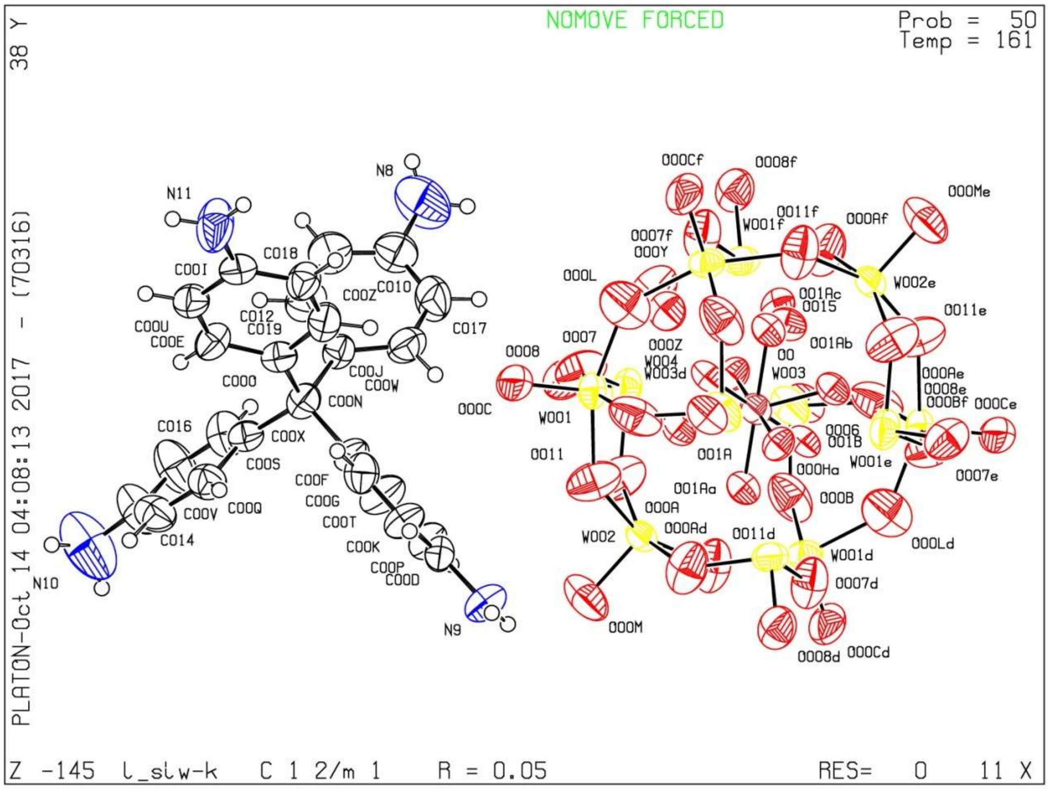

Supplement: Supplementary file 1 [file Data_Sheet_1.PDF]
